# Reinforcement of osteochondoral defects repair with leukocyte platelet-rich fibrin and bone marrow-derived mononuclear cells in a rabbit model

**DOI:** 10.1186/s12891-025-08952-x

**Published:** 2025-07-25

**Authors:** Mohamed Salem, Awad Rizk, Esam Mosbah, Adel Zaghloul, Gamal Karrouf

**Affiliations:** Department of Surgery, Anesthesiology, and Radiology, Faculty of Veterinary Medicine, Mansoura University, Mansoura, 35516 Egypt

**Keywords:** PRF, BM-MNCs, Osteochondral defect, Rabbits, Immunohistochemical study, Relative gene expression

## Abstract

**Background:**

The spontaneous healing of the osteochondral defects leads to the formation of fibrous or fibrocartilage tissue that lack normal cartilage characteristics. Therefore, there are different methods were approved for the functional treatment of osteochondral defects including, microfracture osteochondral mosaicoplasty, autologous chondrocyte implantation, platelets-rich plasma (PRP), bone marrow-derived mononuclear cells (BM-MNCs), Mesenchymal stem cells (MSCs) and platelets-rich fibrin (PRF**).** The present study evaluate the regeneration of osteochondoral defects in rabbits using PRF and BM-MNCs through immunohistochemical (IHC) and gene expression of collagen type II and aggrecan in the regenerated tissue at 3, 6 and 12 weeks postoperative.

**Methods:**

A total of 48 adult male New Zealand white rabbits, aged 5–6 months and weighed 3.5 to 4.0 kg, were used in this study and divided into four experimental groups, where all animals received an osteochondral defect of a 4 mm diameter and 5 mm depth was made in the trochlear groove of the left stifle joints. The defects were left for spontaneous repair in group A. They were filled either with 1 cm^3^ of PRF in group B, 6000k of BM-MNCs in group C or a combination of 0.8 cm^3^ of PRF and BM-MNCs in group D.

**Results:**

Gross observation of the defect, based on the degree of defect repair, the integration to border zone and the appearance of the defect area, was significantly higher in group D than other experimental groups (*P* ≤ 0.05). Microscopical evaluation including surface architecture, tissue morphology, cell distribution and safranin O staining of the matrix was significantly higher in group D than other groups (*P* ≤ 0.05). IHC staining showed a high concentration of collagen type II in groups B and D respectively; a moderate to high amount in groups and a moderate amount in group A (2.0 ± 0.5). The relative gene expression showed a significant increase of collagen type II and aggrecan in group D compared to other groups at all-time points.

**Conclusions:**

The current study’s findings show that when PRF and BMNCs are combined, osteochondral lesions mend more quickly, and the regenerated tissue has stronger collagen type II and proteoglycan deposition than when either substance is utilized alone. To gather proof of the positive benefits of the combination of PRF and BMNCs, more research on clinically afflicted cases is required. Also, Autologous PRF is capable of stimulating BMSC growth and has good biocompatibility and can aid in the restoration of cartilage and subchondral bone.

**Supplementary Information:**

The online version contains supplementary material available at 10.1186/s12891-025-08952-x.

## Introduction

Hyaline articular cartilage is avascular and aneural tissue composed of chondrocytes (1%) embedded in an extracellular matrix (ECM) that is composed of water (65% to 80), collagen and proteoglycan. Collagen type II is predominant in ECM with smaller amounts of other collagen types I, IV, VI, IX, X, and XI [1]. The collagen gives the cartilage its mechanical structure and tensile strength while the aggrecan gives the articular cartilage its compressive strength [2]. Successful treatment of osteochondral defects remains a chief challenge for Orthopaedic clinicians because failure in osteochondral defect repair finally resulting in degenerative joint disease [3, 4].

Cartilage defects are either chondral or osteochondral depending on whether they extend or not to the subchondral bone. Partial defects look like clefts and fissures detected through the early phases of osteoarthritis and do not heal spontaneously [5, 6]. While, Full-thickness defects pass all layers of articular cartilage and penetrate the subchondral bone gaining access to the cells that present in the bone marrow cavity including the mesenchymal stem cells(MSCs), and progenitor hematopoietic cells [7].

The spontaneous healing of the osteochondral defects leads to the formation of fibrous or fibrocartilage tissue that lack normal cartilage characteristics [8]. Therefore, there are different methods were approved for the functional treatment of osteochondral defects including, microfracture [9], osteochondral mosaicoplasty [10], autologous chondrocyte implantation [11], platelets-rich plasma (PRP), BM-MNCs [12], MSCs [13, 14] and PRF [15].

PRF consists of a 3D fibrin network rich in transforming growth factors (TGFs); vascular endothelial growth factors; platelet-derived growth factors (PDGFs), fibroblast growth factors (FGFs), epidermal growth factor (EGF) and insulin-like growth factors (IGFs) that stimulate the growth of cells, the translation of proteins essential for articular cartilage repair [16]. It also facilitates cytokine enmeshment and stem cell migration and is an effective scaffold for the migration and differentiation of bone marrow cells for cartilage or bone regeneration [13, 17–19].

BM-MNCs are a heterogeneous group of cells consisting of varying proportions of differentially matured B cells, T cells, monocytes and progenitor cells including hematopoietic stem cells, mesenchymal stem cells, and endothelial progenitor cells, which have been reported to support regenerative properties against biological stresses [20, 21]. BM-MNCs is rich in chondrogenic proteins that aid in BM-MNCs’ differentiation into chondrocyte [22].

Collagen type II and aggrecan play an important role in the hyaline cartilage function making them the most essential markers for hyaline cartilage differentiation and its expressions pattern in the repaired tissue reflect its similarity to the native hyaline cartilage [23]. The data regarding the use of PRF in combination with BM-MNCs for the treatment of osteochondral defect repair is scarce. Therefore, the present study aimed to evaluate the use of PRF and BM-MNCs in the treatments of osteochondral defects in rabbits as an experimental model based on the evaluation of the quantity of collagen type II and aggrecan through immunohistochemistry and gene expression.

## Materials and methods

### Animals

A total of 48 adult male New Zealand white rabbits aged 5–6 months old, weighing 3.5 to 4.0 kg were used in the present study. The animals were obtained from Mansoura experimental research Centre (MERC), Mansoura University, Egypt. Rabbits were acclimatized to laboratory conditions for two weeks before starting of experiment, kept under constant conditions, and provided with a standard diet and water ad-libitum. The study was performed at MERC, Mansoura University, Egypt. The present study was performed according to the ethical approval from the Scientific Research Ethical Committee, Faculty of Veterinary Medicine of Mansoura University (Code: PhD 15) and all procedures in this study were performed following the ARRIVE guidelines.

### Preparation of PRF

Autologous PRF was prepared according to [24]. In brief, 4 ml of blood was withdrawn immediately before surgery from the ear vein in a plain tube without anticoagulant. The samples were immediately centrifuged at 3000 rpm for 10 min using a laboratory centrifuge (80 L Electric centrifuge, China). The PRF located in the middle of the tube was collected and the red blood cells and plasma were discarded.

### Isolation of BM-MNCs

Autologous bone marrow was aspirated from the femur of each rabbit by an 18-gauge biopsy needle under negative pressure (2.5 ml from each). The BM-MNCs were isolated from the bone marrow by the density gradient method [25]. In brief, each sample of the bone marrow (5 ml) was aspirated in a 10 ml disposable syringe containing 500 IU heparin. The samples were mixed gently using a pipette with 10 mL phosphate-buffered saline (PBS). The sample was poured slowly on the wall of a 50 mL falcon tube containing 10 mL ficole (Ficole^®^, Sigma, USA). The sample was centrifuged at 3000 rpm for 30 min then the translucent ring containing mononuclear cells was aspirated by a pipette. The sample was washed two times with PBS by centrifugation at 2000 rpm for 10 min. Discard the filtrate then resuspend the cell pellet in 60 µL of PBS. The viability of cells was evaluated by trypan blue dye exclusion. The number of cells in each sample was counted by a hemocytometer.

### Creation of osteochondral defect and experimental design

Anesthesia was induced by intramuscular injection of Xylazine HcL (5 mg/kg, Xylaject 2%, ADWIA, Cairo, Egypt); ketamine HCL (35 mg/kg, Aneket^®^, 5% (NEON Laboratories Ltd, Mumbai, India) and butorphanol (0.1 mg/kg, Alvegesic, CP. Pharma, Germany). Besides, lumbosacral epidural analgesia was performed using lidocaine HcL (4 mg/kg: Debocaine, 2%, Arab Company for Gelatin and Pharmaceutical industries, Cairo, Egypt) and Tramadol (4 mg/kg: Minpharm, Grünethal, Germany).

The patella was luxated on the lateral side after the arthrotomy of the stifle joint. An osteochondral defect of 5 mm depth and 4 mm width was done in the middle of the trochlear groove by an electric drill with a drilling bit of 3.5 mm diameter. The rabbits were allocated into four groups (*n* = 12) where the defects were left for spontaneous repair in group A, the defects were filled with PRF gel in group B, and 6000k of BM-MNCs in Group C. In group D, the defect was filled with PRF gel and then injected 6000k of BM-MNCs in PRF gel. The cells were injected with the defect placed in an upright position to prevent the cells from falling out and combining with the clotted blood. After completion of the procedure, the patella was returned to its normal anatomic location and the joint capsule, subcutaneous tissues, and skin were routinely sutured immediately to prevent the loss of the cells. Postoperatively, rabbits received intramuscular injection of cefotaxime (50 mg/kg: Cefotax, Eipico, Cairo, Egypt) for 5 days and meloxicam at a dose of 0.3 mg/kg (ADWIA, Cairo, Egypt) for three days.

### Evaluation of the repaired tissue

#### Gross observation

The International Cartilage Repair Society (ICRS) gross evaluation score was used for macroscopical evaluation of the repair tissue based on gross observation [26] at 3, 6 and 12 weeks postoperatively. The degree of osteochondral defect repair was firstly measured by wound probe and then formulated in percentage to record the exact filling rate.

#### Histological evaluation

Animals were sacrificed using an overdose of thiopental sodium anesthesia in dose (150–200 mg/kg) administered by intraperitoneal injection. All animals were anesthetized and made unconscious before euthanasia. A high dose of thiopental sodium was administered during the euthanasia procedure to ensure a quick loss of consciousness and subsequent cardiac arrest.

Pieces of the femoral condyles measuring 6 × 6 × 6 mm^3^ were cut including healthy tissue around the repaired area. The samples were fixed in 10% formalin and decalcification of tissue was achieved by using EDTA decalcifying solution in distilled water. Each specimen was stained with Hematoxylin and Eosin (H&E) and Safranin O (SO). Histological scoring for the repair of cartilage defect was assessed according to the ICRS scale [27] at 3, 6 and 12 weeks postoperatively.

#### Immunohistochemical staining of collagen type II

The IHC staining for the harvested femoral condyle was done according to [28]. In brief, the sections were deparaffinized with xylene and dissolved in ethanol. Sections were treated with 0.1% pronase enzyme and incubated in Hydrogen Peroxidase Block for 10 min. Apply primary antibody enhancer (Monoclonal anti-rabbit type II collagen antibody) and incubate it at room temperature for 10 min. The defects were scored (0–3) and evaluated at 3, 6, and 12 weeks postoperative. Score 0 indicated no IHC stain; Score 1 means slight positive IHC stain; score 2 means moderate positive IHC stain while score 3 indicates strong positive IHC stain for collagen type II [27].

#### Gene expression of chondrogenesis-related genes

Total RNA from regenerated tissue was extracted using trizol reagent (Thermo Fisher Scientific, UK) according to manufacturer’s instructions. One microgram of extracted RNA was transferred into cDNA using a cDNA synthesis kit (Bioneer kit, K-210). A newly formed cDNA strand was used as a template for gene expression analysis using primer pairs listed in Table (3). PCR was done by 700 Real-Time PCR Systems (Applied Biosystems, Singapore). All PCR reactions were in the following condition: initial 95ºC for 15 min; followed by 35 cycles at 95ºC for 15 s and 60ºC for 30 s. Tubulin was used for internal control. The translation of messenger RNA proteins was calculated following the method of livak and schmittgen using the 2^− ^^ct^ for the calculation of fold change in relation to the control group (Table 1).

**Table 1:** The sequences of primers used in real-time PCR analyses for gene expression of collagen II and Aggrecan

Primer Name	Primer (5`−3`) NCBI	Accession number
House keeping (Tubuline)	Forward: TAGCCAGATCGTGTCCTCCA Reverse: GCACGCTTGGCATACATCAG	NM_001195806.1
Collagen type II	Forward: CCTGTGCGACGACATAATCTGT Reverse: GGTCCTTTAGGTCCTACGATATCCT	AF027122
Aggrecan	Forward: GCTACGGAGACAAGGATGAGTTC Reverse: CGTAAAAGACCTCACCCTCCAT	L38480

### Statistical analysis

The normality of qualitative values (collagen type II and aggrecan mRNA expression and IHC scoring) was assessed using normal probability plots and the Kolmogorov- Simonov test generated with the UNIVARIATE procedure of SAS. All experimental data are expressed as mean ± standard deviation (STD). To assess the effect of PRF and BM- MNCs on gene expression, one-way analysis of variance (ANOVA) was used to analyse the data followed by Tukey-Kramer HSD for multiple comparisons. Statistical analyses were carried out using a commercial program (JMP, version 5.0.1a). Results were considered significant when *P* ≤ 0.05.

## Results

### Gross examination

#### At three weeks

**Fig. 1:**
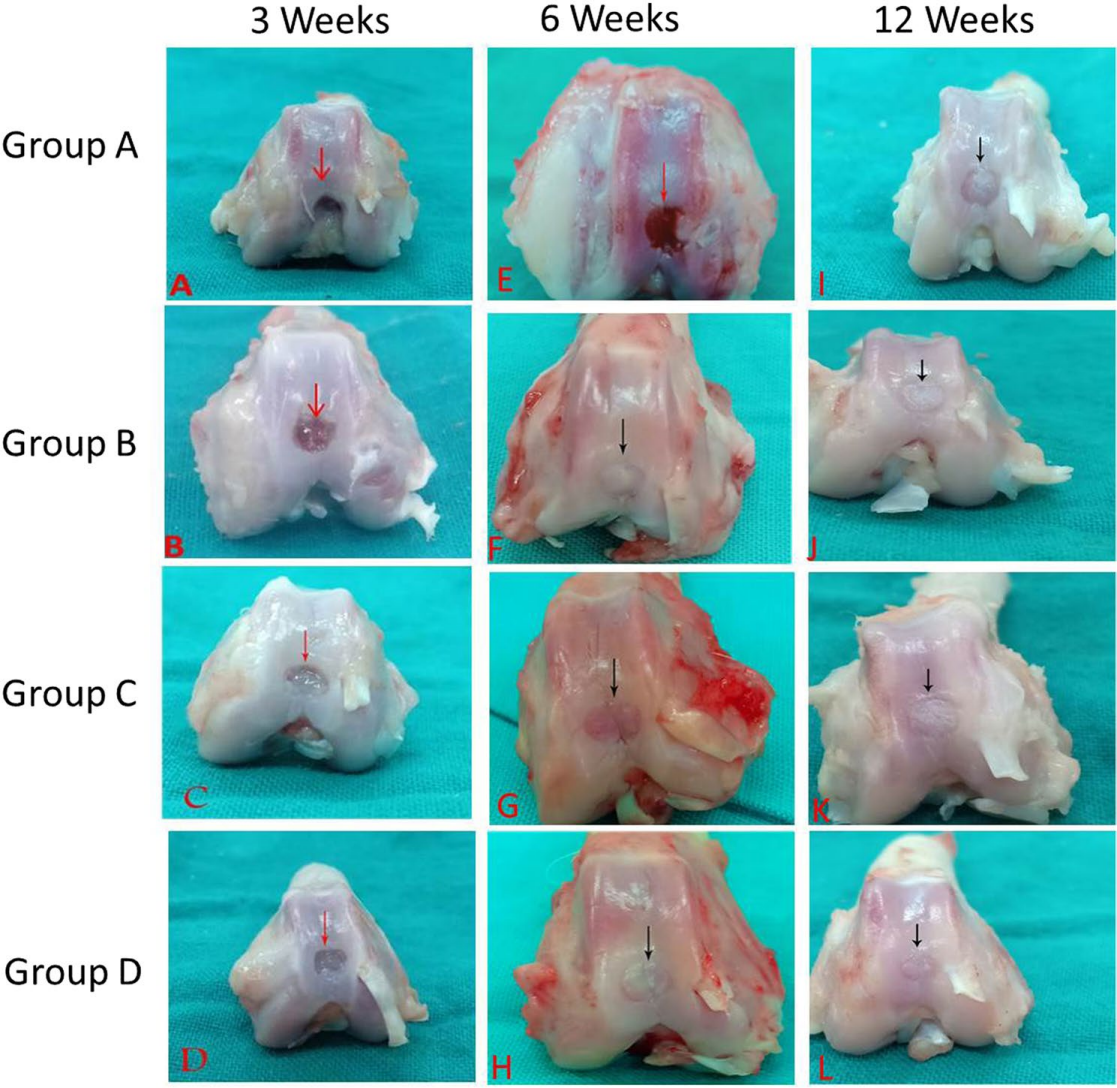
Gross appearance of the osteochondral defects in trochlear groove of left femur in New Zealand White rabbits at 3, 6 and 12 weeks postoperative. Gross appearance showed better healing in group D followed by groups B, C, and A respectively in terms of degree of defect repair; Integration to border zone; Appearance of defect area; colouration of defect area, Defect area congestion; Group A: Control Group, Group B: PRF, Group C: MNCs, Group D: PRF and MNCs

**Fig. 2:**
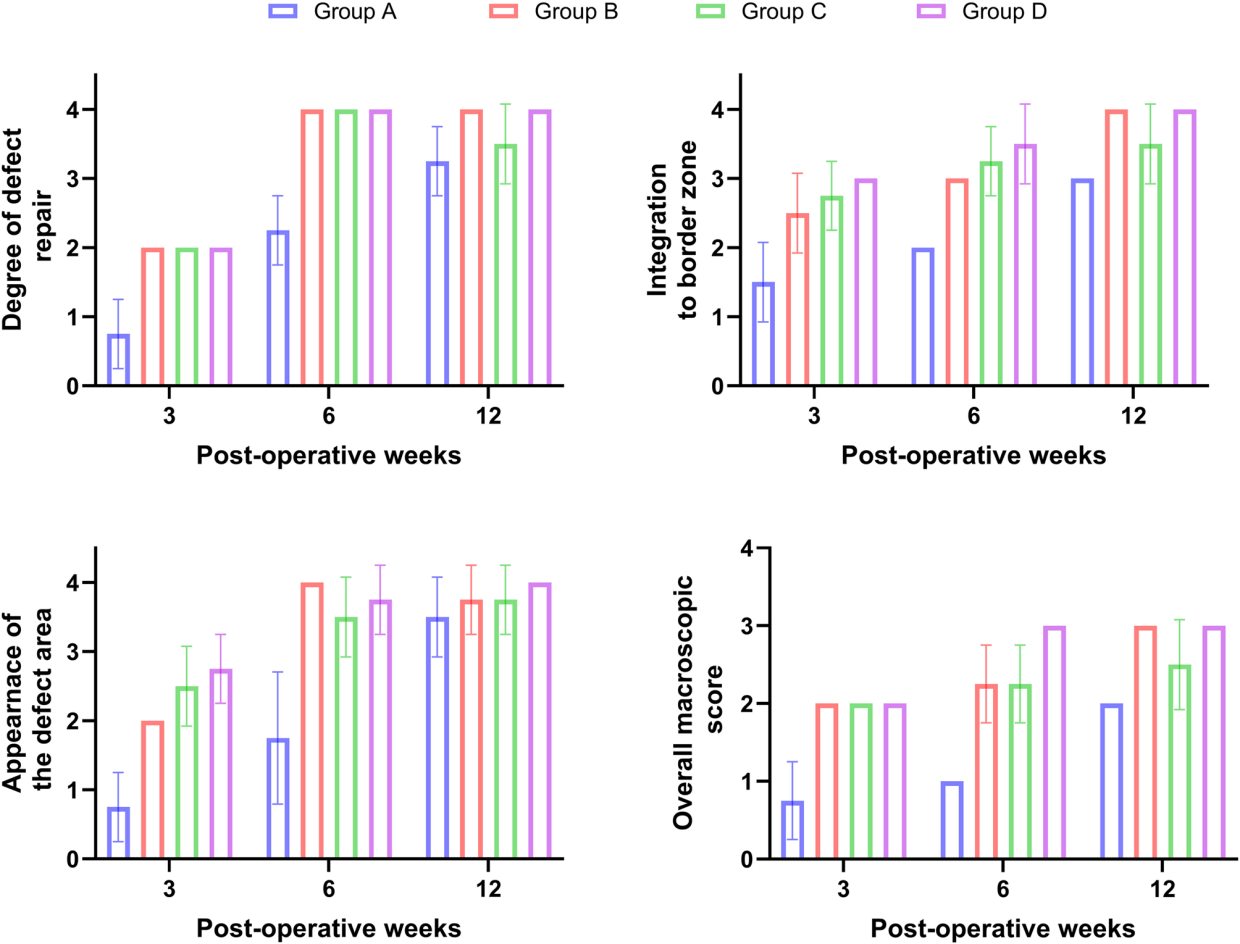
Charts of the ICRS gross evaluation score at 3, 6 and 12 weeks postoperative showed significant increase in the degree of defect repair, integration to border zone, appearance of the defect area and overall gross observation score in group D compared with group A B and C. Group A: Control Group, Group B: PRF, Group C: MNCs, Group D: PRF and MNCs

The osteochondral defects were filled by 25% of its depth in the animals of group A (control; 0.6 ± 0.5); 50% in groups B (PRF; 1.8 ± 0.5); 25 to 50% in group C (BM-MNCs; 1.5 ± 0.5), and 50 to 75% in group D (PRF and BM-MNCs; 2.0 ± 0.0). The integration to the border zone showed a significant increase in group D (3.0 ± 0.0) compared with groups A, B and C (1.5 ± 0.6; 2.5 ± 0.6 and 2.0 ± 0.0) respectively. The defect area had several large fissures in group A (0.8 ± 0.5) while it had small, scattered fissures and cracks in groups B and C (2.70.5and2.0 ± 0.0) and fibrillated surface in group D (3.0 ± 0.0). The color of the newly formed tissue varied from yellowish opaque to reddish appearance in the control group (0.5 ± 0.6) while it had a whitish opaque appearance in groups B and C (2.3 ± 0.0 and 2.0 ± 0.0), respectively, and transparent appearance with a whitish ring in group D (3.0 ± 0.0). The overall repair assessment at 3 weeks suggested grade IV in group A (0.8 ± 0.5) and grade III in groups B, C and D (2.0 ± 0.0; 1.5 ± 0.0; 2.0 ± 0.0) respectively (Figs. 1 and 2).

#### At six weeks

The osteochondral defects were filled by 50% of their depth in rabbits of group A (2.2 ± 0.5) while they were filled by 75% in group B (3.7 ± 0.5); 50 to 75% of their depth in group C (2.5 ± 0.5), and 75 to 100% in group D (3.8 ± 0.2). Integration to border zone showed a significant increase in group D (3.8 ± 0.2) compared with groups A, B and C (2.0 ± 0.0; 3.5 ± 0.5 and 2.3 ± 0.5) respectively. The defects appeared to have small, scattered fissures or cracks in groups A and C (1.8 ± 0.9 and 3.0 ± 0.0), fibrillated surface in group B (3.7 ± 0.5) and intact smooth surface in group D (4.0 ± 0.0). The colour of the healing tissue was reddish in groups A and C (2.0 ± 0.0 and 3.1 ± 0.4) respectively, transparent appearance with a whitish ring in group B (3.3 ± 0.5) while it has a transparent appearance in group D (4.0 ± 0.0). The overall repair assessment at six weeks was categorized as abnormal healing (grade III) in group A (2.0 ± 0.0), grade II in the animals of group C (3.0 ± 0.0), and grade I (normal healing) in groups B and D (3.6 ± 0.5 and 3.8.0 ± 0.2) respectively (Figs. 1 and 2).

#### At twelve weeks

The osteochondral defects were filled up to 75% in animals of group A (3.0 ± 0.0), 75 to 100% in group C (3.2 ± 0.5) and 100% in group B and D (3.8 ± 0.5 and 4.0 ± 0.0) respectively. Integration to border zone showed a significant increase (*P* < 0.05) in group D (4.0 ± 0.0) compared with groups A, B, and C (3.0 ± 0.0; 3.8 ± 0.6 and 3.2 ± 0.5) respectively. The defect area appeared to have tiny, scattered fissures in group A (3.1 ± 0.6), fibrillated surface in group C (3.5 ± 0.5) and an intact smooth surface in groups B and D (3.8 ± 0.6 and 4.0 ± 0.0) respectively. The color of the healed tissue was transparent with a whitish ring in groups A and C (2.8 ± 0.0 and 3.5 ± 0.5) respectively and transparent appearance in animals of groups B and D (3.8 ± 0.5 and 4.0 ± 0.0) respectively. The overall repair assessment at 12 weeks revealed grade II in groups A and C (3.0 ± 0.0 and 3.3 ± 0.5) respectively and grade I in groups B and D (3.8 ± 0.5and 4.0 ± 0.0) respectively (Figs. 1 and 2).

### Microscopical findings

#### At three weeks

**Fig. 3:**
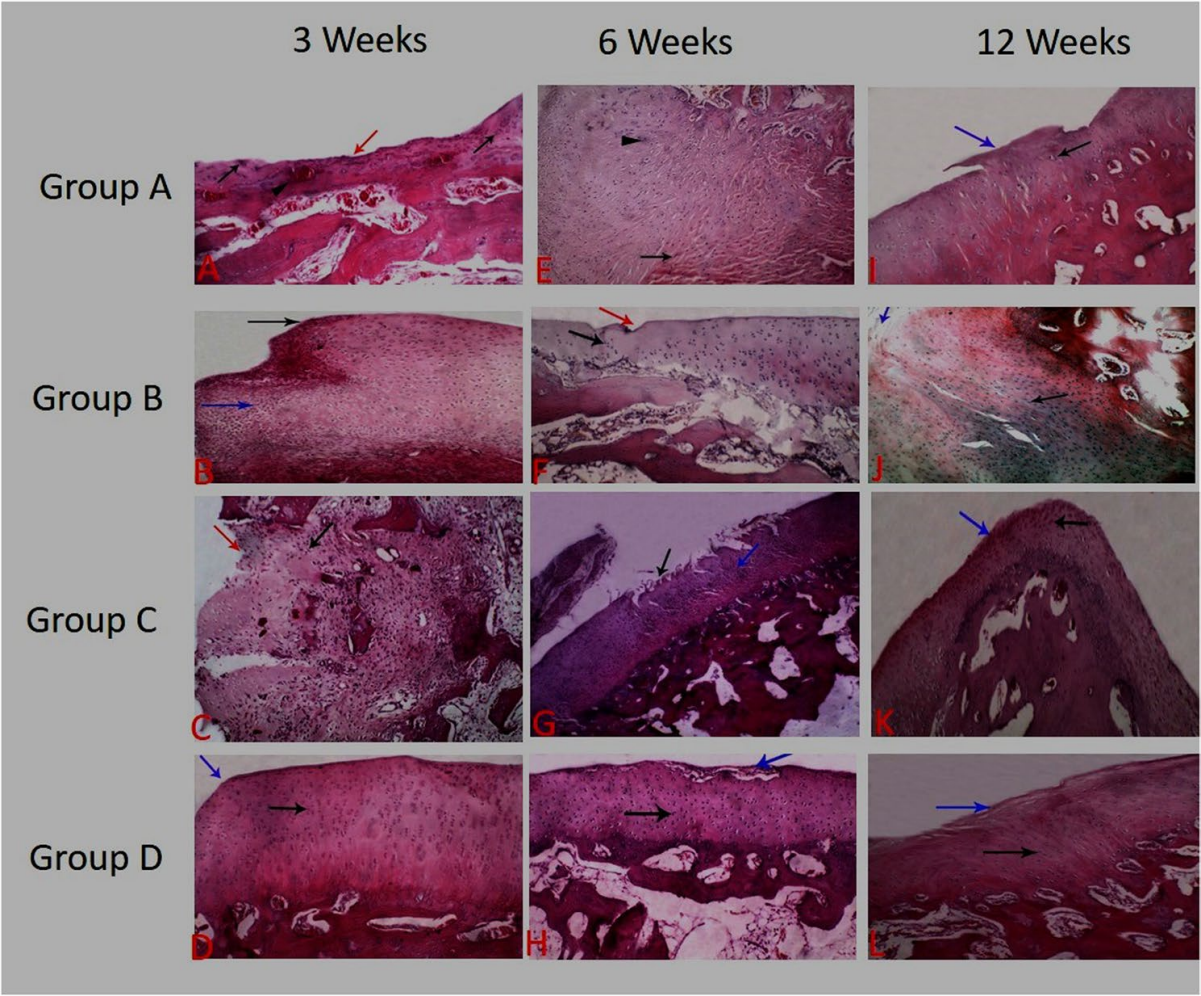
Histopathological view of osteochondral defect at trochlear groove of left stifle joint of white New Zealand rabbit at 3, 6 and 12 weeks postoperative A, Erosion of articular surface with exposing subchondral bone (red arrow) with aggregation of chondrocytes in the form of clusters at the edges of osteochondral defect (black arrows) and congestion of subchondral capillaries (arrow head) in group A at 3 week. B, Proliferation of the fibrocartilagenous tissue filling the osteochondral defect (black arrow) characterized disorganized chondrocytes in the form of clusters and columnar, and spindle shaped fibroblasts (blue arrow) in group B at 3 week. C, Fibrocollagenous tissue (red arrow) filling the osteochondral defect characterized by aggregated chondrocytes and spindle shaped fibroblasts in group C (black arrow) at 3 weeks. D, Smooth incontinuous surface (blue arrow)with fibrocartilage filling the osteochondral defect characterized by mixed columnar and clustered shaped chondrocytes (black arrow) in group D at 3 week.E, Exuberant proliferation of fibrocartilagenous tissue filling the osteochondral defect (arrow) and aggregation of chondrocytes in the form of clusters at the edges (arrow head) in group A at 6 weeks. F, Displays smooth incontinuous surface (red arrow) with hyaline cartilage filling the osteochondral defect characterized by columnar shaped chondrocytes (black arrow) in group B at 6 week. G showed Cartilage fibrillation with irregular surface (black arrow) with fibrocartilage filling the osteochondral defect characterized by mixed columnar and clustered shaped chondrocytes (blue arrow) in group C at 6 week. Figure H showed Smooth incontinuous surface (blue arrow) with hyaline cartilage filling the osteochondral defect characterized by columnar shaped chondrocytes (black arrow) in group D at 6 week. Figure I showed: Fibrocartilagenous tissue filling the osteochondral defect (blue arrow) characterized by hypocellualrity and aggregation of chondrocytes in the form of clusters (black arrow) in group A at 12 week. Figure J showed Smooth continuous surface (blue arrow) with hyaline cartilage filling the osteochondral defect characterized by columnar shaped chondrocytes (black arrow) in group B at 12 week. figure K showed Smooth continuous surface (blue arrow)with mixed hyaline and fibrocartilage cartilage filling the osteochondral defect characterized by mixed columnar and clustered shaped cells (black arrow) in group C at 12 week. Figure L showed Smooth continuous surface (blue arrow)with hyaline cartilage filling the osteochondral defect characterized by columnar shaped chondrocytes (black arrow) in group D at 12 week. Group A: Control Group, Group B: PRF, Group C: MNCs, Group D: PRF and MNCs

**Fig. 4:**
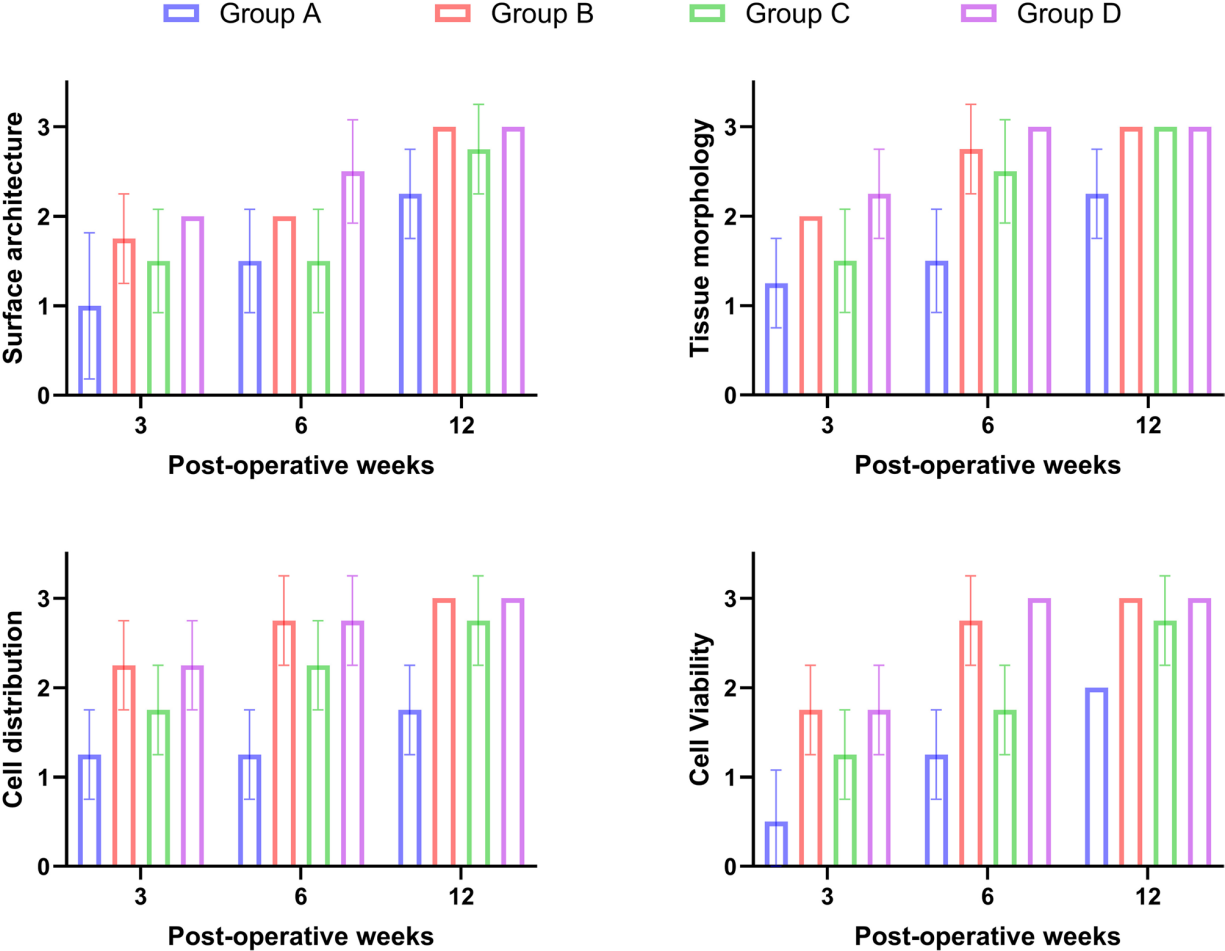
Chart of the ICRS histological score at 3, 6 and 12 weeks postoperative showing significant increase in tissue morphology, tissue architecture, cell distribution and cell viability in group D compared with other groups. Group A: Control Group, Group B: PRF, Group C: MNCs, Group D: PRF and MNCs

The repaired tissue in animals of the control group contains fibrous tissue with severe congestion (0.3 ± 0.6). The tissue architecture was irregular with disorganized cells, erosion of the articular surface and exposing subchondral bone (0.3 ± 0.5). The cell population viability was less than 10% (0.3 ± 0.5) with mild to moderate calcification (0.8 ± 0.5). The mean score of cell distribution in healed tissue was (0.8 ± 0.5). The overall repair assessment suggested grade III in the control group (0.8 ± 0.5). While in group B (PRF), the repaired tissue contains fibrocartilage (2.3 ± 0.0). The tissue architecture was smooth and discontinuous (1.8 ± 0.5). The cells were arranged in the repaired tissues in the form of mixed columnar and cluster manner (2.3 ± 0.5) with population viability of 75% (2.0 ± 0.0) without any calcification (Figs. 3 and 4).

The repaired tissue of group C (BM-MNCs) contains fibrocartilage (1.3 ± 0.5) with a moderately irregular surface (1.5 ± 0.5). The cells were arranged in mixed columnar and cluster shapes (1.8 ± 0.5) with population viability was about 50% (1.8 ± 0.5) without any calcification. While group D (PRF and BM-MNCs), the regenerated tissue contains mixed hyaline and fibrocartilage (2.5 ± 0.5) with smooth discontinuous surface architecture (2.0 ± 0.0). The arrangement of cells was mixed columnar and clusters (2.5 ± 0.5) with population viability of 75% (2.3 ± 0.5) without any calcification (Figs. 3 and 4).

**Fig. 5:**
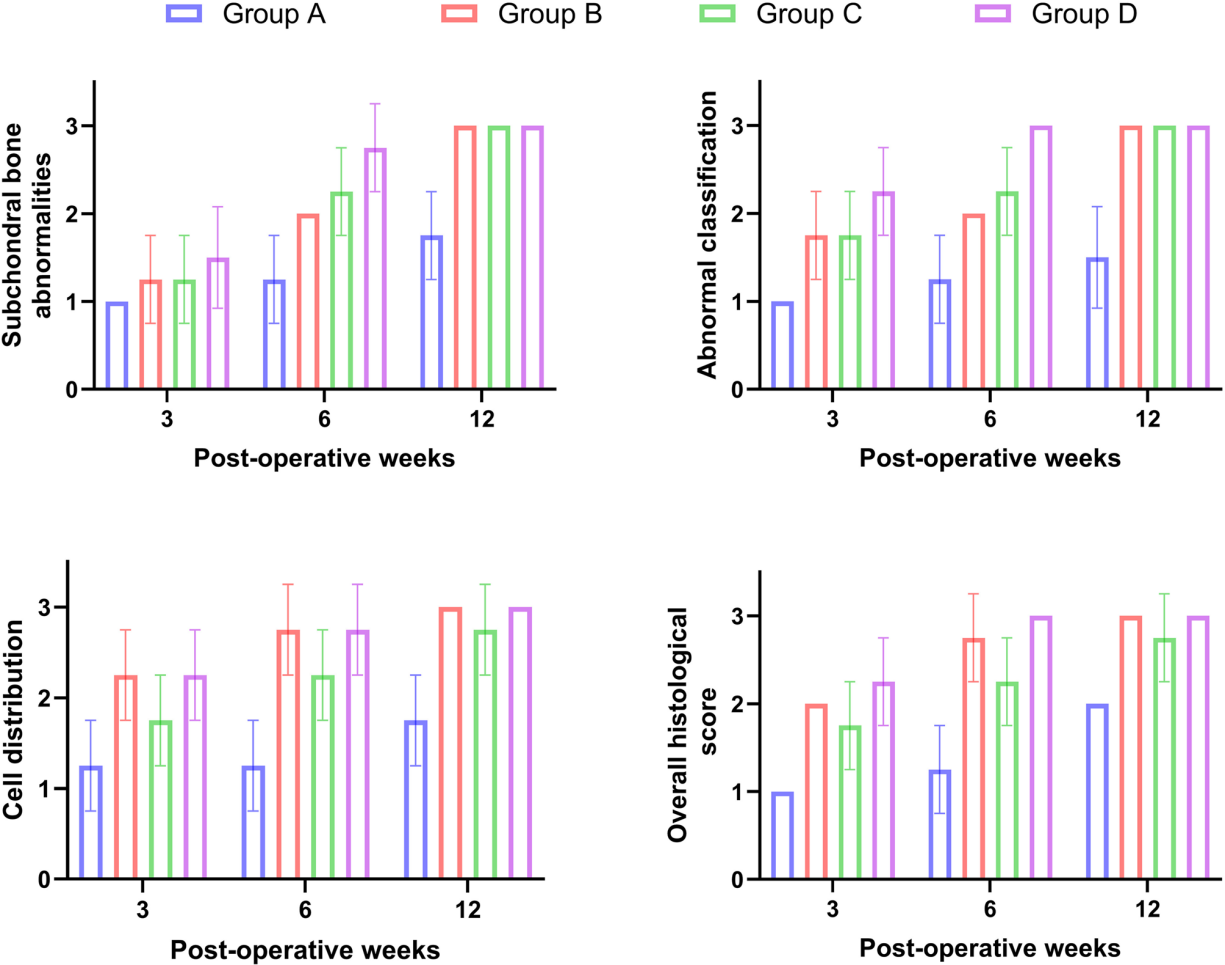
Chart of the ICRS histological score at 3, 6 and 12 weeks postoperative showing significant increase in the score of subchondral bone abnormalities, abnormal calcification, safranin O staining and overall histological evaluation in group D compared with other groups. Group A: Control Group, Group B: PRF, Group C: BMNCs, Group D: PRF and BMNCs

**Fig. 6:**
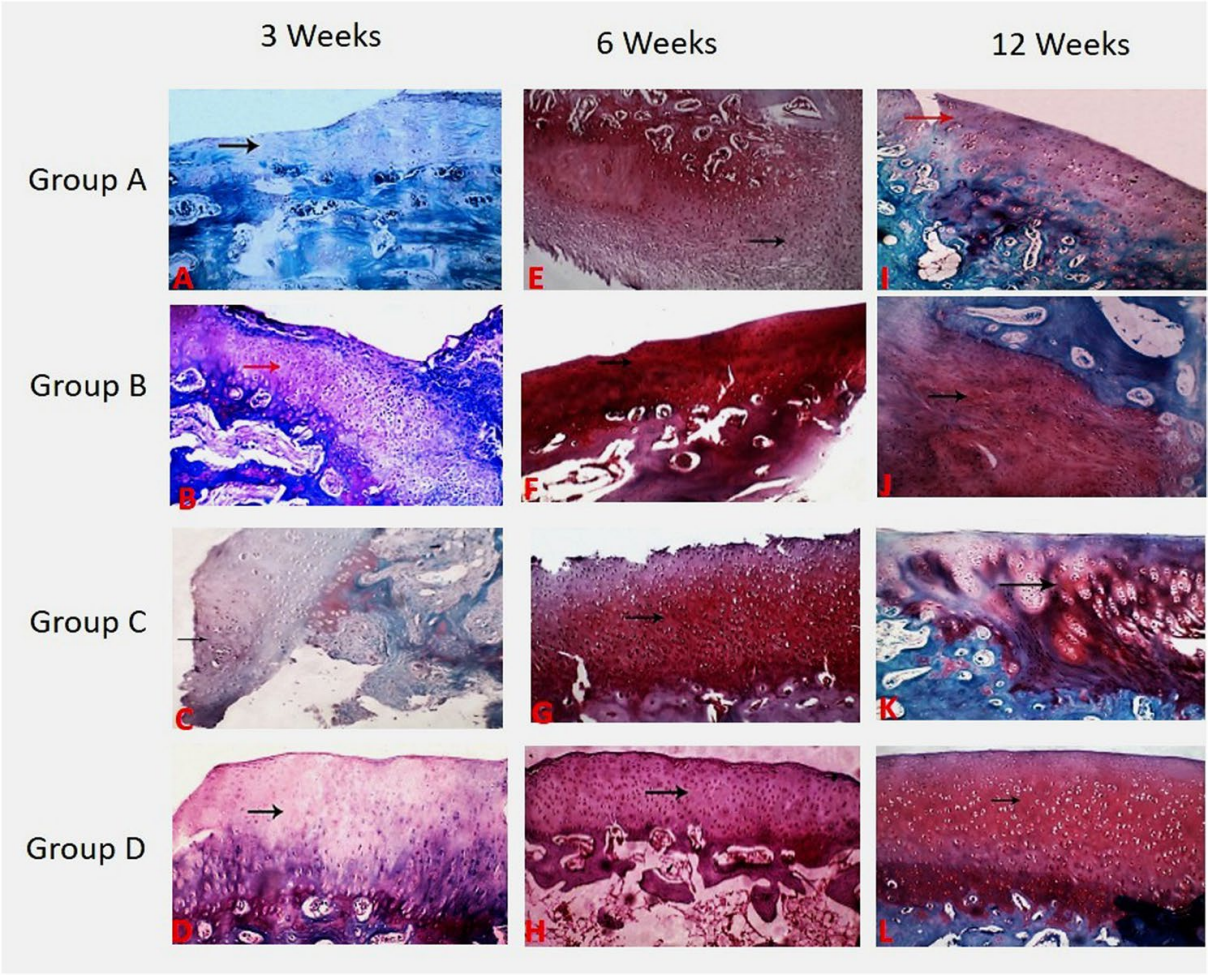
Histopathological view of osteochondral defect at trochlear groove of left stifle joint of white New Zealand rabbits at 3, 6 and 12 weeks postoperative showed normal matrix staining by safranin O (red color) and chondrocytes arrangement in group D followed by groups B, C, and A respectively (safranin O stain, 100x).Group A: Control Group, Group B: PRF, Group C: BMNCs, Group D: PRF and BMNCs

While Safranin O staining of repaired tissue in the control group showed the absence of the red colour of safranin O stain (0.0 ± 0.0) specific for chondroid matrix. While group B, it showed a moderate red-chondroid matrix (1.8 ± 0.5) with safranin O stain. In group C, there was a slight red-stained chondroid matrix (1.5 ± 0.5) with safranin O stain. In group D, a moderate red-stained chondroid matrix (2.3 ± 0.0) was detected with safranin O stain. The overall repair assessment at 3 weeks suggested grade III in group A (0.8 ± 0.5) while grading II (nearly normal) in groups B, C and D respectively (2.0 ± 0.0; 1.8 ± 0.5; 2.3 ± 0.5) (Figs. 5 and 6).

#### At six weeks

The healed tissue in the control group contains fibrous tissue with blood vessels and exuberant proliferation of fibrocartilagenous tissue filling the osteochondral defect (1.0 ± 0.5). The tissue architecture was moderately irregular (1.2 ± 0.5). The cell population viability was 50% (0.8 ± 0.5) with mild to moderate calcification (1.0 ± 0.0). The cells in the repaired tissues were arranged in clusters (1.0 ± 0.0). While in group B, the repaired tissue contains hyaline cartilage (2.5 ± 0.5) with a smooth and discontinuous surface (2.0 ± 0.0) and columnar-shaped cell arrangement (2.5 ± 0.5). The cell population viability was predominantly viable (2.5 ± 0.5) (Figs. 3 and 4).

The healed tissue in the BM-MNCs group contains fibrocartilage cartilage (2.0 ± 0.0). The tissue architecture was moderately irregular with cartilage fibrillation (1.5 ± 0.6) and characterized by mixed cluster and columnar cell arrangement (2.3 ± 0.5) While in Group D, the regenerated tissue contains hyaline cartilage (3.0 ± 0.0) with smooth and discontinuous surface (2.5 ± 0.6**).** The cell population viability was predominantly viable (3.0 ± 0.0) with columnar-shaped cell arrangement (2.8 ± 0.5) (Figs. 3 and 4). Safranin O Staining of regenerated tissue showed a slight red-chondroid matrix in group A (0.8 ± 0.5), a moderate red-chondroid matrix in group C (2.0 ± 0.0) and a normal red-stained chondroid matrix with safranin O stain in groups B and D (2.5 ± 0.5 and 3.0 ± 0.0) respectively. The overall repair assessment at 6 weeks suggested grade III in group A (1.0 ± 0.0), grade II in group C (2.0 ± 0.0) while grade I (nearly normal) in groups B and D (2.8 ± 0.5; 3.0 ± 0.0) respectively (Figs. 5 and 6).

#### At twelve weeks

The healed tissue in animals of the control group contains mixed fibrocartilage (2.3 ± 0.5) with smooth and discontinuous surface (2.3 ± 0.5) and mixed columnar and clusters cells arrangement (0.1 ± 0.0). The cell population viability was about 75% (2.0 ± 0.0) without any calcification. While in group B, the repaired tissue contains hyaline cartilage (2.8 ± 0.5) with smooth and continuous tissue architecture (3.0 ± 0.0). The cell population viability was predominantly viable (2.7 ± 0.5) with columnar-shaped cell arrangement in the repaired tissues (2.7 ± 0.5). The repaired tissue showed complete incorporation with subchondral bone (Figs. 3 and 4).

The repaired tissue in rabbits of the BM-MNCs group showed mixed hyaline and fibrocartilage (2.5 ± 0.5) with smooth and continuous tissue architecture (2.8 ± 0.5). The cell population viability was 75% (2.3 ± 0.5). The cells were arranged in the repaired tissues in mixed columnar and clusters shape (2.5 ± 0.5). While in group D, the regenerated tissue contains hyaline cartilage (3.0 ± 0.0) with smooth and continuous surface and columnar-shaped cell (3.0 ± 0.0). The cell population viability was predominantly viable (3.0 ± 0.0). Safranin O staining of regenerated tissue in the control group showed a slight red-chondroid matrix (1.8 ± 0.5), a moderate red-chondroid matrix in group C (2.5 ± 0.5), and a normal red-stained chondroid matrix with safranin O stain in groups B and D **(**2.8 ± 0.5 and 3.0 ± 0.0) respectively. The overall repair assessment at 12 weeks suggested grade II in groups A and C (2.0 ± 0.0 and 2.3 ± 0.5) respectively while grade I (nearly normal) in groups B and D (2.8 ± 0.3 and 3.0 ± 0.0) respectively (Figs. 5 and 6).

### Immunohistochemistry findings

**Fig. 7:**
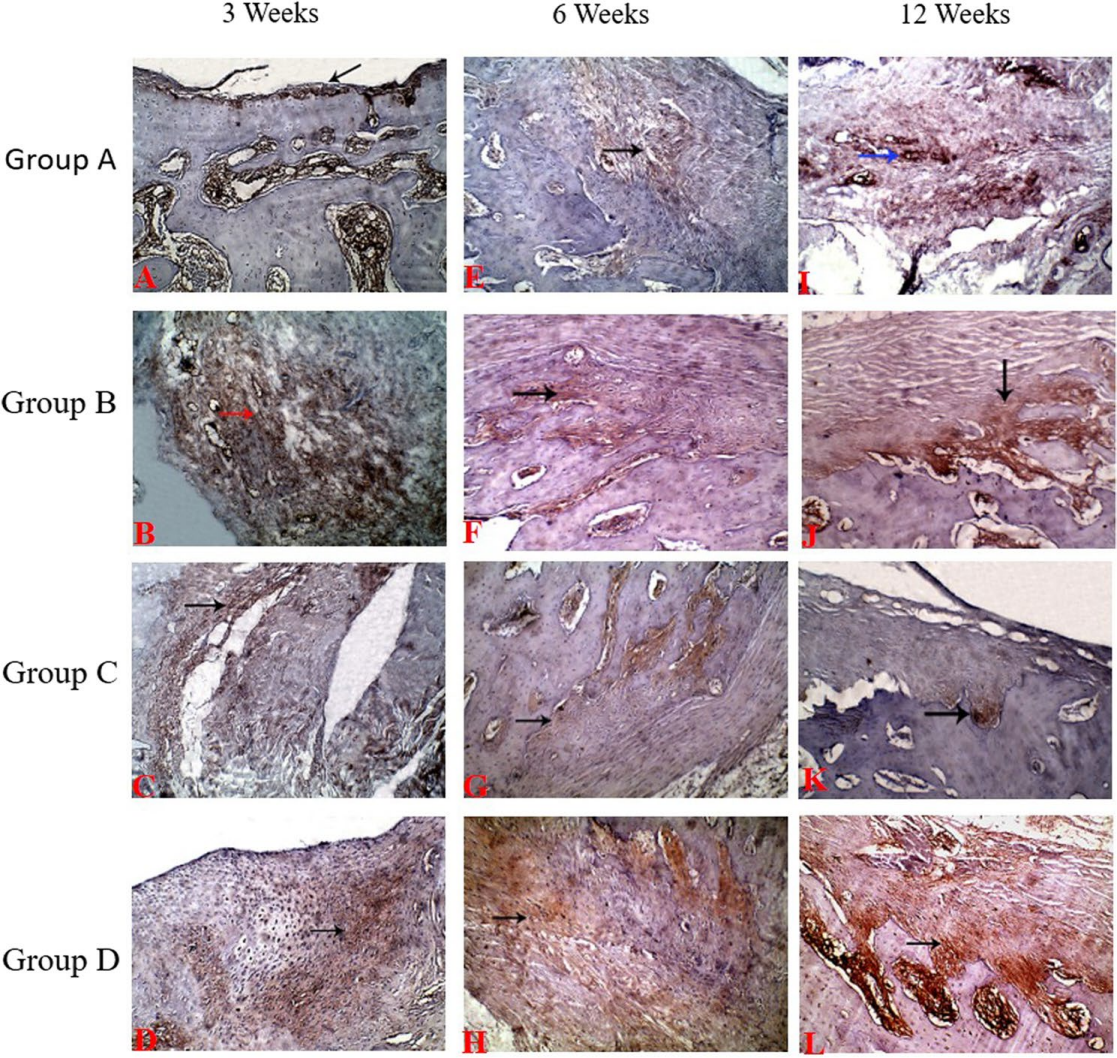
Immunohistochemical view of the defects at 3, 6 and 12 weeks postoperative (IHC, 100x). Group A: Control Group, Group B: PRF, Group C: BMNCs, Group D: PRF and BMNCs

At three weeks postoperative, the repaired tissue in group A showed a negative reaction for anti-collagen II (0.0 ± 0.5) indicated by the absence of brownish discoloration in the matrix (Fig. 7A) while it showed slight positive brown immunostaining for anti-collagen II in group C (1.3 ± 0.5) (Fig. 7C) and moderate positive brown immunostaining B and D (1.8 ± 0.5 and 2.3 ± 0.5) respectively (Fig. 7B and D). At six weeks, it showed mild positive brownish immunostained coloration (0.8 ± 0.5) against collagen type II in group A (Fig. 7E) while there was strong positive brownish immunostained coloration against collagen II in groups B and D (2.5 ± 0.5; and 3.0 ± 0.5) respectively (Fig. 7F and H) and moderate positive brownish immunostained coloration against collagen II in group C (2.0 ± 0.5) (Fig. 7G). At twelve weeks, the repaired tissue in group 1 showed moderate positive brownish immunostained coloration (2.0 ± 0.5) against collagen II (Fig. 7I); moderate to high positive brownish immunostained coloration (2.3 ± 0.5) in group C (Fig. 7K) while it showed strong positive brownish immunostained coloration against collagen II in group B, and D (2.8 ± 0.5and 3.0 ± 0.5) respectively (Table 4; Fig. 7J and L).

### Effect of PRF and BM-MNCs on the expression of collagen type II and aggrecan gene

**Fig. 8:**
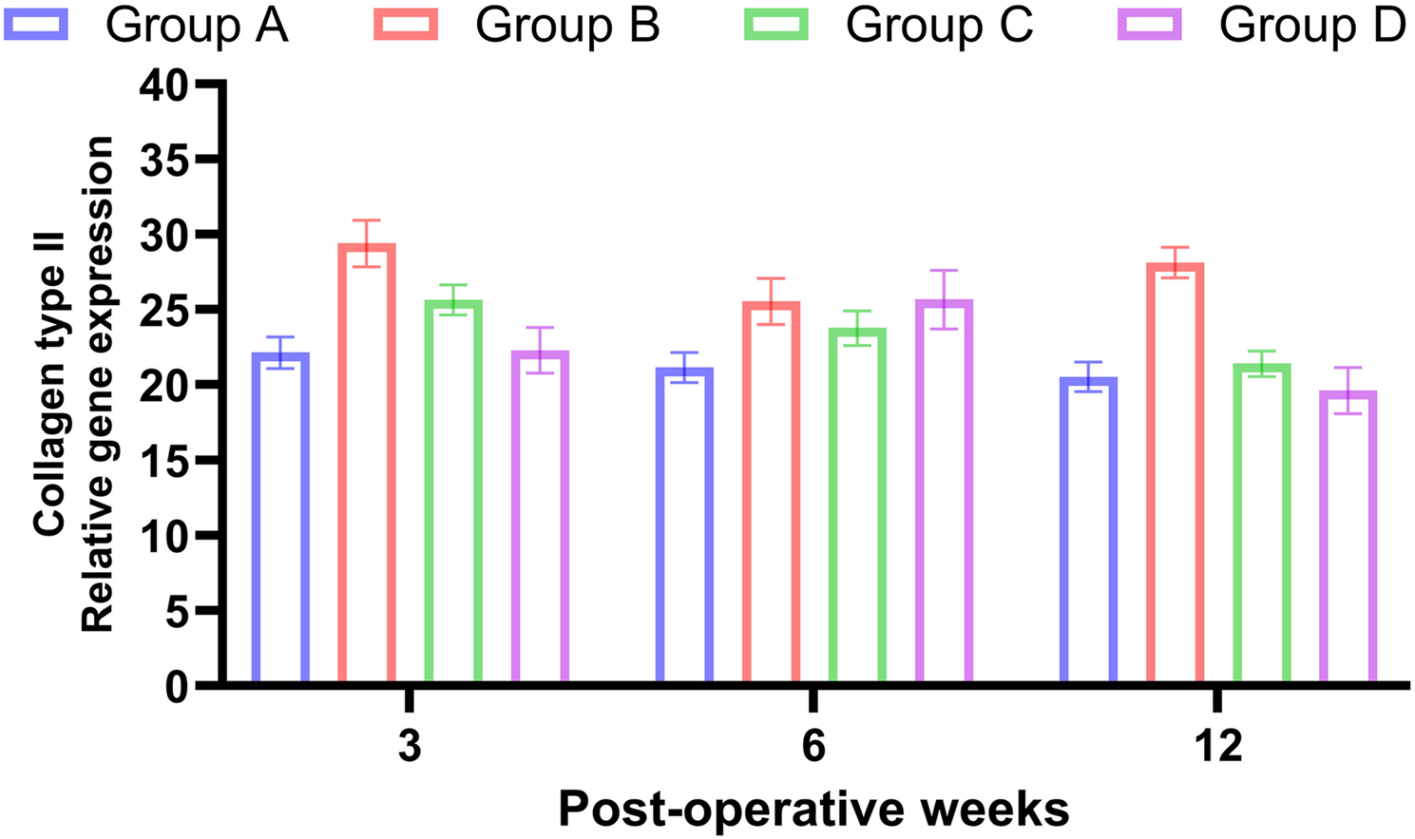
Chart illustrates the relative gene expression of Collagen type II at 3, 6 and 12 weeks postoperative in regenerated tissue following repair of osteochondoral defect in stifle joint of rabbits. Group A: Control Group, Group B: PRF, Group C: BMNCs, Group D: PRF and BMNCs

The expression of mRNA for collagen type II was significantly up-regulated at all-time points in PRF and BM-MNCs combination group when compared to other groups [3 weeks, 6.1-fold; 6 weeks, 10.1 fold; 12 weeks, 14.1-fold, *P* < 0.05]. In the PRF group, the fold changes were also significantly increased when compared to sham-operated rabbits **[** 3 weeks, 4-fold; 6 weeks, 8.1 fold; 12 weeks, 12.5 fold, *P* < 0.05**].** Meanwhile, in the BM-MNCs group, there were no significant changes from the sham-operated group (*p* > 0.05) at 3 weeks postoperative. Collagen type II mRNA was significantly induced at 6 weeks (4.1-fold) and 12 weeks (9.1 fold) compared with the sham-operated group (Table 2; Fig. 8).

**Table 2:** Showed mean ± standard deviation of Type-II collagen ICH staining of the matrix during osteochondoral defect repair in stifle joint of rabbits. Group A: Control, Group B: PRF, Group C: BMNCs, Group D: PRF and BM-MNCs

Groups	Time post treatment		
	3 weeks	6 weeks	12 weeks
Group A (*n* = 12)	0.0 ± 0.5	0.8 ± 0.5	2.0 ± 0.5
Group B (*n* = 12)	1.8 ± 0.5	2.5 ± 0.5	2.8 ± 0.5
Group C (*n* = 12)	1.3 ± 0.5	2.0 ± 0.5	2.3 ± 0.5
Group D (*n* = 12)	2.3 ± 0.5	3.0 ± 0.5	3.0 ± 0.5

**Fig. 9:**
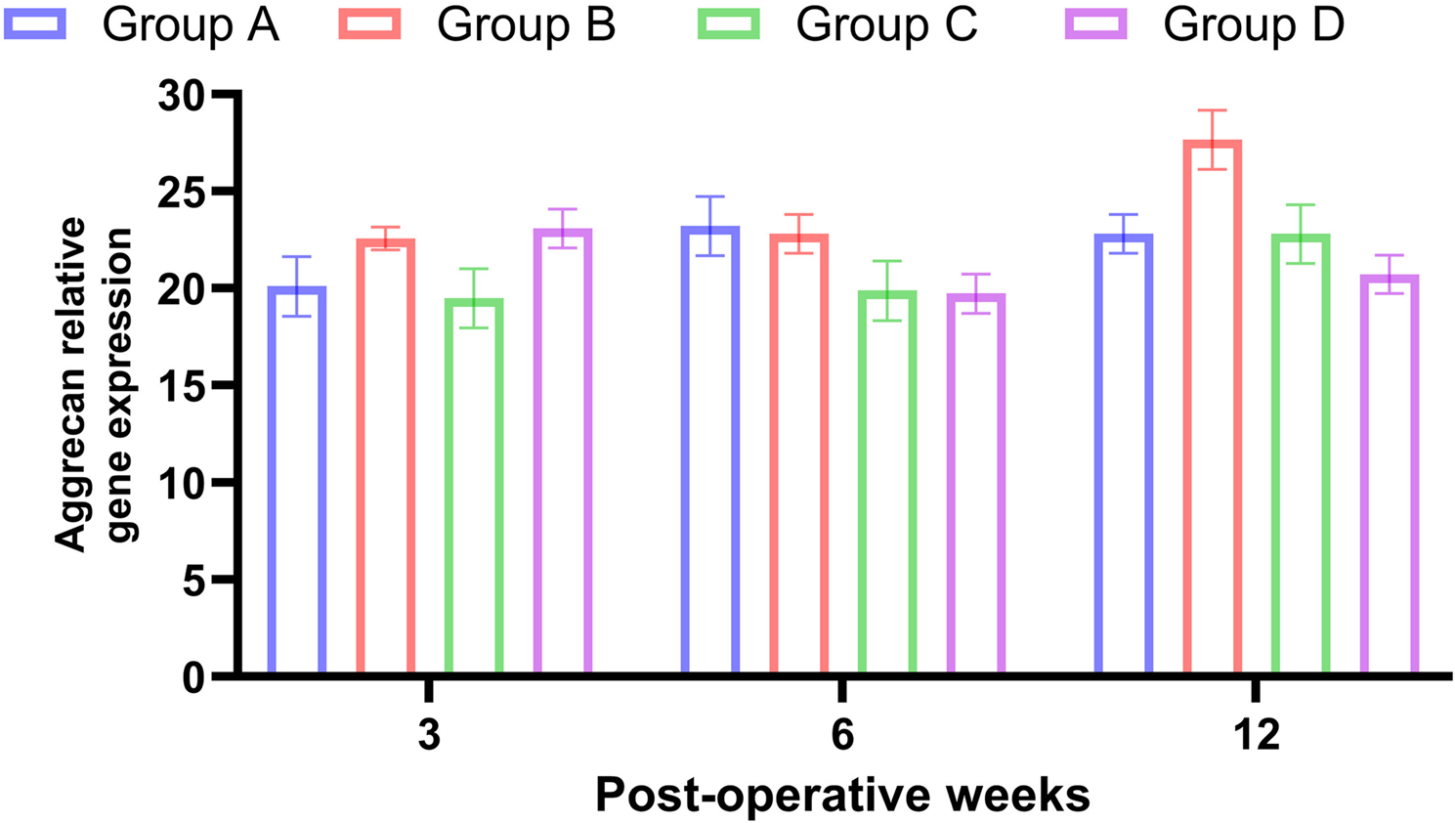
Chart illustrates the relative gene expression of aggrecan at 3, 6 and 12 weeks postoperative in regenerated tissue following repair of osteochondoral defect in stifle joint of rabbits. Group A: Control Group, Group B: PRF, Group C: BMNCs, Group D: PRF and BMNCs

The expression of aggrecan was significantly higher at all times of evaluation in PRF and BM-MNCs combination group when compared to other treatments [3 weeks, 6.5 fold; 6 weeks, 10.3 fold; 12 weeks, 15.5 fold, *P* < 0.05]. While in the PRF group, the fold changes were also significantly higher when compared to the control group [3 weeks, 5.4-fold; 6 weeks, 7.7 fold; 12 weeks, 11.4 fold, *P* < 0.05]. The fold change of aggrecan was higher in the BM-MNCs group in comparison with the control group [3 weeks, 3.2 fold; 6 weeks, 5.6-fold; 12 weeks, 8.2-fold, *P* < 0.05] (Table 3; Fig. 9).

**Table 3:** Showed mean ± standard deviation of fold change of relative collagen type II expression in the regenerated tissue following osteochondral defect repair in stifle joint of rabbits. Group A: control, Group B: PRF, Group C: BM-MNCs, Group D: PRF and BM-MNCs

Groups	Time post treatment		
	3 weeks	6 weeks	12 weeks
Group A (*n* = 12)	1.0 ± 0.05	1.0 ± 0.05	1.0 ± 0.05
Group B (*n* = 12)	4.0 ± 0.01	8.1 ± 0.01	12.5 ± 0.01
Group C (*n* = 12)	1.5 ± 0.02	4.1 ± 0.02	9.1 ± 0.02
Group D (*n* = 12)	6.1 ± 0.01	10.1 ± 0.01	14.1 ± 0.01

## Discussion

Articular cartilage has the inadequate ability for repair due to a lack of blood and nerve supply. As soon as it is injured, it is usually healed with fibrous tissue, especially in the elderly which is devoid of the characteristics of the normal cartilage [8]. The treatment options for injured cartilage are limited and the traditional ones are not ideal [29]. Osteochondral defects are still a challenging clinical problem because these defects were could not be restored spontaneously leading to biological regeneration for osteoarthritic joints [7]. An osteochondral defect of 5 mm depth and 4 mm width was performed at the trochlear groove of the femur. Defects of similar shape and size have been used earlier for osteochondral defect repair studies [12, 15]. In the current study, an osteochondral defect was performed in the trochlear groove of the femur to decrease stress at the defect location as great stress has been stated to encourage fibrous tissue formation and suppress chondrogenesis and hyaline cartilage formation [30]. Rabbits are useful for the evaluation of osteochondral defect repair because easy handling, economic, have a short healing time and have larger joints suitable for surgical handling [31].

PRF was used for the repair of osteochondral defect due to the richness by many growth factors including IGF, TGF-b and FGF which increase chondrocyte metabolism and differentiation, proliferation, formation of extracellular matrix and do not make an immunological reaction or carcinogenic effects with the low financial cost for preparation [32, 33]. In addition, it does not make an immunological reaction or carcinogenic effects with the low financial cost for preparation. PRF has extra advantages over PRP in that PRF is produced with a single centrifugation procedure and contains a higher concentration of growth factors [15, 34].

Previous studies have shown that the effectiveness of bone marrow cells is often limited by two serious factors: the lower survival capacity of the implanted cells due to the severe acidic, hypoxic and avascular microenvironment at the defect site, and lack of control over their differentiation into the terminal cell types, which is needed to replace the lost tissues [35]. Meanwhile, platelet growth factors such as IGF and TGF-b are known to stimulate bone marrow cells proliferation, reduce apoptosis and induce expression of chondrogenic markers and ECM deposition [36], so the use of PRF in combination with BM-MNCs may help to overcome both of these therapeutic challenges.

The defects in the control group have impaired filling rate and appearance at 3 weeks postoperative. Histologically, the repaired tissue contains fibrous tissue with severely irregular tissue architecture, erosion of articular surface and disorganizing cells. These findings were in coincidence with [20] who observed hypocellularity, disorganization, proteoglycan reduction, fissures and fibrillation of articular cartilage at the defect of non-treated animals. Fissures that occur in non-treated osteochondral defects are due to the reduction of proteoglycans which disturb the balance between the internal swelling pressure of the proteoglycan and the tension of type II collagen fibres [37]. Moreover, hypocellualrity occurred in the control group due to apoptosis and chondrocyte aggregation in large lacuna with the presence of fibrous connective tissue bridging across the fissures. Also, matrix formation by these cells is troubled by shifting the metabolism towards catabolic effects by producing matrix-degrading enzymes such as proteases, aggrecanase or collagenase which can split the collagen fibres and help in breaking down the cartilage [38].

The negative safranin O stain of ECM and immunostaining for anti-collagen II in the control group at 3 weeks postoperative indicate the absence of collagen II fibres and the formation of fibrous tissue. These results were in agreement with [39] who mentioned, negative immunostaining against collagen type II in the non-treated articular defects. This is due to the absence of collagen type II that considered the main component of articular cartilage due to failure of hyaline cartilage production.

In the present study, the degree of defect repair showing a significant increase in group D in comparison with other groups at 3 weeks postoperative. Moreover, this repaired tissue is mostly mixed hyaline and fibrocartilage in group D while it contains fibrous tissue with severe congestion in the control group, and fibrocartilage in PRF and BM-MNCs groups. The better filling rate enhanced by better tissue morphology in PRF and BM-MNCs combination group over other groups is due to the interaction between the implanted cells that contain MSCs and progenitor cells with PRF scaffold that contain chondrogenic growth factors that help in differentiation of the these cells into chondrocyte with the acceleration of ECM production [13, 40].

In the current study, the score of appearance of the defect area is a significant increase in the combination group than in other groups. The newly repaired tissue has fibrillated surface in group D and small scattered fissures and cracks in groups B and C. By routine stain, these fibrillated surfaces in group D appeared to have a smooth discontinuous surface architecture compared with a moderately irregular surface in BM-MNCs group [12, 15, 41]. The regenerated tissue was superior in PRF and BM-MNCs combination group in term of defect appearance and histologically tissue architecture due to synergistic action between the implanted cells with PRF scaffold as PRF act as an excellent bioactive scaffold rich in chondrogenic growth factors that provide a suitable media for sticking, growth and differentiation of BM-MNCs into chondrocyte and therefore the production of hyaline ECM [15, 42].

In the present study, the result of Safranin O staining showed a slight red-stained chondroid matrix at BM-MNCs, a moderate amount of red-stained chondroid matrix in groups B and D at 3 weeks postoperative [15, 20]. The better safranin O staining and ECM deposition in the regenerated tissue in PRF and PRF with BM-MNCs combination are attributed to that, PRF matrix has the capacity to trap circulating cytokines and cells [24], and therefore their lifespan is increased which improves their usage in the primary stages of matrix remodeling moreover, PRF facilitate the homing and differentiation of bone marrow cells into chondrocyte [13, 43].

In the present investigation, the regenerated tissue showed slight positive brown immunostaining in BM-MNCs treated group and moderate positive brown immunostaining in B and D groups. These results are attributed to that, transplanted cells can discriminate into chondrocytes and assist in osteochondral defect repair [12]. In addition, transplanted BM-MNCs and PRF matrix can secrete some cytokines such as TGF-b which aid in differentiation of bone marrow cells into chondrocytes [12, 20, 21, 40].

The relative expression of collagen type II at 3 weeks postoperative showed significant increase in group D (6.1-fold) compared to groups A, B and C (1, 4, 1.5-fold) respectively. This is explained by transplanted mononuclear cells can secrete cytokines such as TGF-b which aid in differentiation of bone marrow cells into chondrocytes [22] moreover, PRF is rich in chondrogenic growth factors as TGF-b and IGF that suppressed IL-1ß that induced cartilage degradation moreover, EGF stimulate the chondrocyte to produce collagen type II and aggrecan [15, 44].

Histologically, the healed tissue in control group at 6 week contains fibrous tissue with blood vessels and exuberant proliferation of fibrocartilagenous tissue filling the osteochondral defect. These results were in agreement with [45] who observed fibrous tissue with shrunken chondrocytes, irregular and condensed nucleus surrounded by vacuolated and intensified stained cytoplasm and arranged in clusters shape in the non-treated rabbits. This is attributed to that, there are few numbers of stem cells from the subchondral bone fail to stick to the defect site and differentiate into chondrocyte with failure of hyaline tissue production.

At the end of the sixth week, the osteochondral defects were filled by 50% of its depth in rabbits of control group while it filled by 75% in PRF group 50 to 75% of its depth in BM-MNCs group, and 75 to 100% in combination group with high significant integration to the border zone. This newly formed tissue in combination group contains hyaline cartilage with smooth surface [13, 20, 40]. This could be explained as mentioned by [20] that transplanted BM-MNCs contained T cells, B cells, monocyte in addition to MSCs, on stimulation, these cells might secrete some cytokines such as TGF-β, which aid in differentiation of BM-MNCs into chondrocyte. In addition, when these cells combined with PRF that rich with osteogenic and chondrogenic growth factors, the differentiation of these cells and ECM production are increased [13].

Grossly, the defect areas appeared to have small, scattered fissures or cracks in group A and C, fibrillated surface in group B and intact smooth surface in group D. Histologically, the regenerated tissue in group D has high cell population viability with columnar shaped cell arrangement compared with moderately irregular tissue architecture with cartilage fibrillation and cell viability 75% in BM-MNCs group at 6 weeks postoperative. Similar finding were obtained by [12, 40, 46].

Safranin O staining of regenerated tissue showed slight red-chondroid matrix in control group, moderate red-chondroid matrix in group C and normal red-stained chondroid matrix with safranin O stain in groups Band D, respectively. These superior results in tissue morphology, architecture, and ECM deposition in PRF containing groups attributed to that, PRF can regulate residual chondrocytes and MSCs from subchondral bone or synovial fluid for cartilage regeneration. Also, PRF is rich in TGFs, IGFs, PDGFs, and EGF. IGF-1 and PDGF suppressed IL-1b-induced cartilage degradation [47]. Moreover; the roles of TGF-b encompass differentiation and de-differentiation of chondrocytes, and synthesis of collagen and proteoglycans to maintain homeostasis of the cartilage [44].

Mild positive brownish immunostained colorations were recorded in the control group while it was moderate in group C and strong in group B, and D respectively at 6 weeks postoperative. These finding could be attributed to exogenous source of BM-MNCs and MSCs found in the transplanted cells and the interaction between transplanted BM-MNCs with normal as well as damaged chondrocytes at osteochondral defects and in synovial fluid, as chondrocytes might have been liberated in synovial fluid during creation of osteochondral defect. Moreover, the injured chondrocytes may release different cytokines that could encourage better proliferation as well as differentiation of BM-MNCs and progenitor cell into chondrocyte [12, 22].

The relative gene expression of collagen type II showed significant increase in group D (10.1-fold) compared to groups A, B and C (1, 8.1, 4.1-fold) respectively. While the relative gene expression of aggrecan showed significant increase in group D (10.3-fold) compared to groups A, B and C (1, 7.7, 5.6-fold) respectively at 6 weeks postoperative. The relative gene expression of collagen type II and aggrecan in group of PRF and BM-MNCs combination is higher than PRF or BM-MNCs alone due to that; PRF is rich in TGFs, b-FGF, IGFs, and EGF. IGF-1 and PDGFs suppressed IL-1ß that induced cartilage degradation moreover, EGF stimulate the implanted cells differentiation into chondrocyte and produce extracellular matrix [15, 44].

At the end of the study, the regenerated tissue in group B and D contain mostly hyaline cartilage with smooth and continuous surface and columnar shaped cells compared with mixed hyaline and fibrocartilage cartilage and little cell viability in control and BM-MNCs group. These results could be attributed to that, implantation of BM-MNCs cells alone in repair of articular Cartilage defect was the problem of homing and sticking to the defect site [20] but when combined with PRF, it gives more better effect because PRF act as bioscaffold that has the ability to trap bone marrow cells and aid in its homing and differentiation into chondrocyte and therefore increase the production of ECM especially Collagen type II and proteoglycans [13, 48].

The additional use of PRF to BM-MSCs transplants in an osteochondral defect studied by [49] that found that PRF significantly improved functional scores of the knee joint within 6 months as assessed by MRI. This may be attributed to the increased concentration of platelets, leukocytes and growth factors that initiates the migration and proliferation of chondroblasts and BMSCs at early stages of cartilage regeneration [50].

Grossly, the defect areas appeared to have tiny, scattered fissures in group, fibrillated surface in group C and intact smooth surface in groups B and D respectively at 12 weeks postoperative. Histologically, the repaired tissue in control and BM-MNCs groups has smooth and discontinuous surface and mixed columnar and clusters cells arrangement, smooth and continuous tissue architecture in groups B and D. These results were attributed as mentioned by [12, 15, 48] to that, the transplanted BM-MNCs contain monocyte, MSCs, TGF β and hematopoietic stem and progenitors cells in high amount (6 × 10^6^) and could be differentiated into chondrocyte. Moreover, PRF act as 3 D bioscaffold rich in several growth factors trap these cells aiding in its homing and discrimination into chondrocyte in high amount than when these cells are used alone.

Also, the repaired tissue has moderate red-stained chondroid matrix in BM-MNCs treated rabbits and high excellent positive safranin O staining in PRF and PRF combined with BM-MNCs with Safranin O staining. The strong safranin O staining in groups B and D is due to excellent expression of proteoglycan produced due to the high differentiation of BM-MNCs and MSCs in these groups into chondrocytes that have the ability to produce proteoglycan [13, 15] moreover, IGF, FGF and TGF-b present in PRF stimulates the expression of cartilage matrix genes, resulting in increased synthesis of proteoglycan and collagen type II in the monolayer culture of chondrocytes [51] but in control and BM-MNCs groups, failure of cell differentiation due to failure in cell sticking and homing impaired the ECM production and proteoglycan deposition [20].

The repaired tissue from rabbits treated by BM-MNCs showed moderate positive brownish immunostained coloration against collagen type II; high positive immunostaining of collagen type II in PRF and PRF combined with BM-MNCs groups. This finding could be attributed to that, platelet concentrates has the ability to control inflammatory and catabolic environment through its anti-inflammatory properties shifts the macrophage polarization from M1(found during tissue damage) toward M2 (found during tissue repair) phenotype [52].

The relative expression of collagen type II showed significant increase in group D (14.1-fold) compared to groups A, B and C (1, 12.5, 9.1-fold) respectively while the relative expression of aggrecan showed significant increase in group D (15.5 fold) compared to groups A, B and C (1, 11.4, 8.2 fold) respectively at 12 weeks postoperative. This superior expression of ECM components in group D was attributed to, firstly, the exogenous source of BM-MNCs contain MSCs and several types of growth stimulating cytokines that play important role in stimulation the differentiation of these cells into chondrocyte [12, 22]. Secondly, the PRF act as excellent bioscaffold that has the ability to trapping the BM-MNCs, MSCs and damaged chondrocyte and help in their differentiation into chondrocyte by the assistant of its chondrogenic cytokines as TGFβ and IGF [46, 48]. Moreover PRF does not dissolve quickly after implantation because of its unique structural characters and the hard consistency of fibrin which leads to slow discharge of growth factors over prolonged period of time than other platelet concentrates [15, 24].

The overall gross, histological, immunohistochemistry and gene expression of osteochondral defect at all-time points were superior and have higher score in PRF containing groups than other groups and more excellent in the combination group. These excellent results in BM-MNCs and PRF combination group in comparison to other groups are explained by that, PRF can regulate residual chondrocytes and MSCs from subchondral bone or synovial fluid for cartilage regeneration. Also, PRF is rich in IGF-1 and PDGF that suppressed IL-1b- that induces cartilage degradation [47]. The roles of TGF-b encompass differentiation and de-differentiation of chondrocytes, and synthesis of collagen and proteoglycans to maintain homeostasis of the cartilage [44].

PRF has many types of cytokines and growth factors as TGFs, PDGFs, FGFs and IGFs that have the ability to stimulate differentiation and proliferation of cells, increase cell motility, and matrix production by binding to specific receptors on the cell surface [13, 40]. Moreover, injected cells alone were located on the surface of regenerated cartilage [53] but when combined with PRF produce a better therapeutic effect due to PRF being enriched by growth factors that stimulate matrix biosynthesis of articular chondrocytes [13]. Also, PRF could encourage angiogenesis because it has thrombin at a low level that is considered to be ideal for the migration of endothelial cells and fibroblasts moreover, its 3-D fibrin network resulted in the capture of cytokines into its mesh architecture [54].

In conclusion, the expression of collagen type II and aggrecan in the rabbits treated with PRF and BM-MNCs combination is higher than in other treated groups. This evidence indicates the high efficacy of this combination as a novel bioactive material which can be used in regenerative medicine to enhance the treatment of osteochondral defects.

### Study limitation

While our study demonstrated consistent results across macroscopic, histological, Immunohistochemical staining of collagen type II and RT-PCR analyses, we acknowledge the absence of protein-level validation, such as Western blotting, as a limitation. Although the concordance among the applied methods supports the reliability of our findings, the addition of Western blot analysis would have further strengthened the interpretation by confirming gene expression changes at the protein level.

## Supplementary Information

Supplementary Material 1.

## Data Availability

“The datasets used and/or analysed during the current study are available in the manuscript”.

## Abbreviations

BM-MNCs: Bone marrow-derived mononuclear cells
PRF: Platelet-rich fibrin
IHC: Immunohistochemical
ECM: Extracellular matrix
MERC: Mansoura experimental research centre
