# Supplementary material for: Reinforcement of osteochondoral defects repair with leukocyte platelet-rich fibrin and bone marrow-derived mononuclear cells in a rabbit model
Source: BMC Musculoskelet Disord. 2025 Jul 25;26:707. doi: 10.1186/s12891-025-08952-x (PMC12297477; doi:10.1186/s12891-025-08952-x)
Supplement: Supplementary file 1 — Supplementary Material 1. [file 12891_2025_8952_MOESM1_ESM.docx]

| **Primer Name** | **Primer (5`-3`) NCBI** | **Accession number** |
| --- | --- | --- |
| House keeping  (Tubuline) | Forward: TAGCCAGATCGTGTCCTCCA  Reverse: GCACGCTTGGCATACATCAG | NM_001195806.1 |
| Collagen type II | Forward: CCTGTGCGACGACATAATCTGT  Reverse: GGTCCTTTAGGTCCTACGATATCCT | AF027122 |
| Aggrecan | Forward: GCTACGGAGACAAGGATGAGTTC  Reverse: CGTAAAAGACCTCACCCTCCAT | L38480 |

**Table 1:** The sequences of primers used in real-time PCR analyses for gene expression of collagen II and aggrecan.

| Time post treatment | | | Groups |
| --- | --- | --- | --- |
| 12 weeks | 6 weeks | 3 weeks |  |
| 2.0±0.5 ^b^ | 0.8±0.5^d^ | 0.0 ±0.5^d^ | Group A (n=12) |
| 2.8±0.5 ^a^ | 2.5±0.5 ^b^ | 1.8±0.5^b^ | Group B (n=12) |
| 2.3±0.5 ^b^ | 2.0±0.5 ^c^ | 1.3±0.5 ^c^ | Group C (n=12) |
| 3.0±0.5 ^a^ | 3.0±0.5 ^a^ | 2.3±0.5^a^ | Group D (n=12) |

**Table 2:** Showed mean ± Standard Deviation of Type-II collagen ICH staining of the matrix during osteochondoral defect repair in stifle joint of rabbits. Group A: Control, Group B: PRF, Group C: BMNCs, Group D: PRF and BM-MNCs.

| Time post treatment | | | Groups |
| --- | --- | --- | --- |
| 12 weeks | 6 weeks | 3 weeks |  |
| 1.0±0.05 | 1.0±0.05 | 1.0±0.05 | Group A (n=12) |
| 12.5±0.01 | 8.1±0.01 | 4.0±0.01 | Group B (n=12) |
| 9.1±0.02 | 4.1±0.02 | 1.5±0.02 | Group C (n=12) |
| 14.1±0.01 | 10.1±0.01 | 6.1±0.01 | Group D (n=12) |

**Table 3:** Showed mean ± Standard Deviation of fold change of relative collagen type II expression in the regenerated tissue following osteochondral defect repair in stifle joint of rabbits. **Group A:** Control, **Group B**: PRF, **Group C:** BM-MNCs, **Group D:** PRF and BM-MNCs.


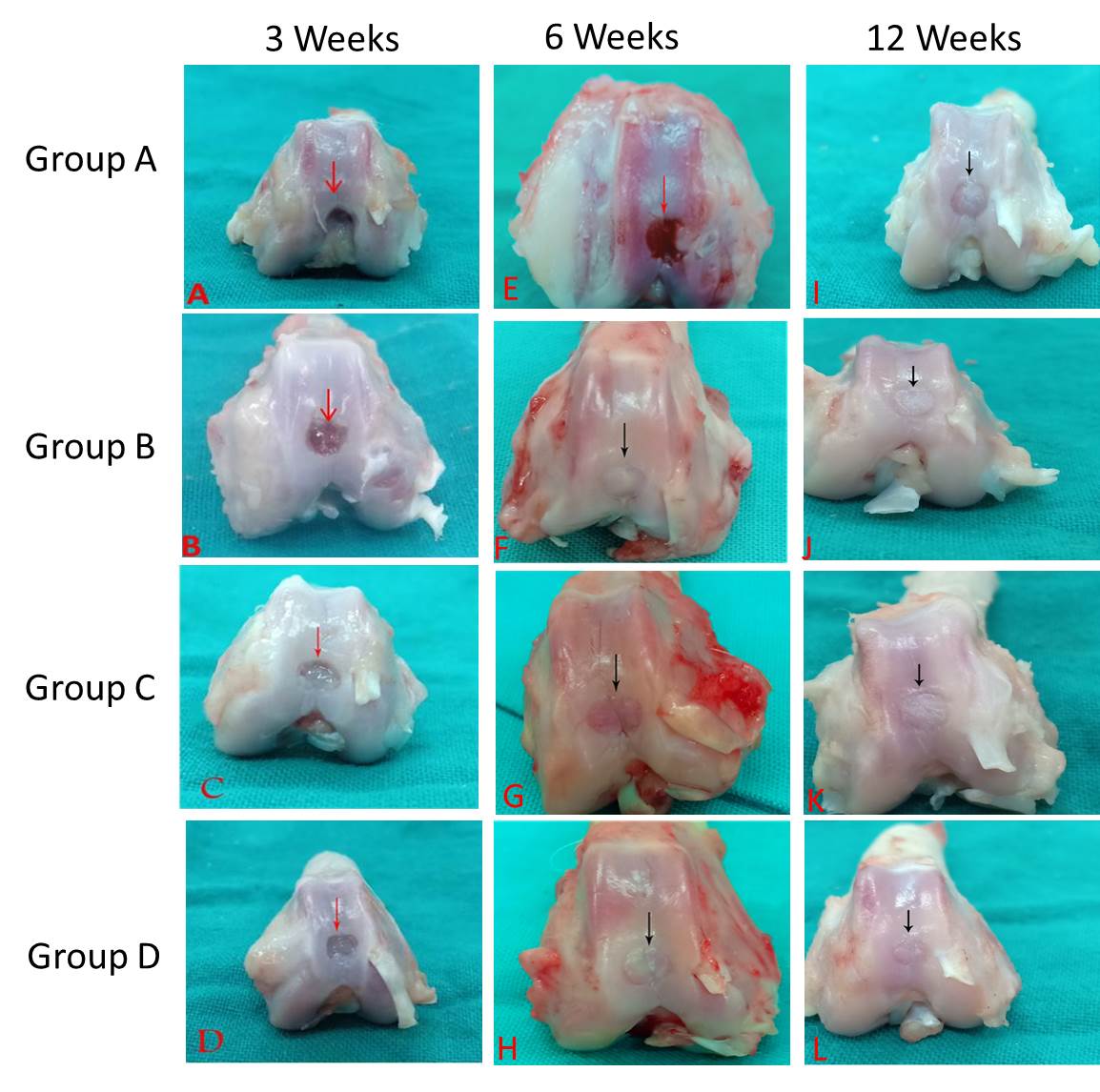


**Figure 1:** Figure 1. Gross appearance of the osteochondral defects in trochlear groove of left femur in New Zealand White rabbits at 3, 6 and 12 weeks postoperative. Gross appearance showed better healing in group D followed by groups B, C, and A respectively in terms of degree of defect repair; Integration to border zone; Appearance of defect area; colouration of defect area, Defect area congestion; Group A: Control Group, Group B: PRF, Group C: MNCs, Group D: PRF and MNCs.


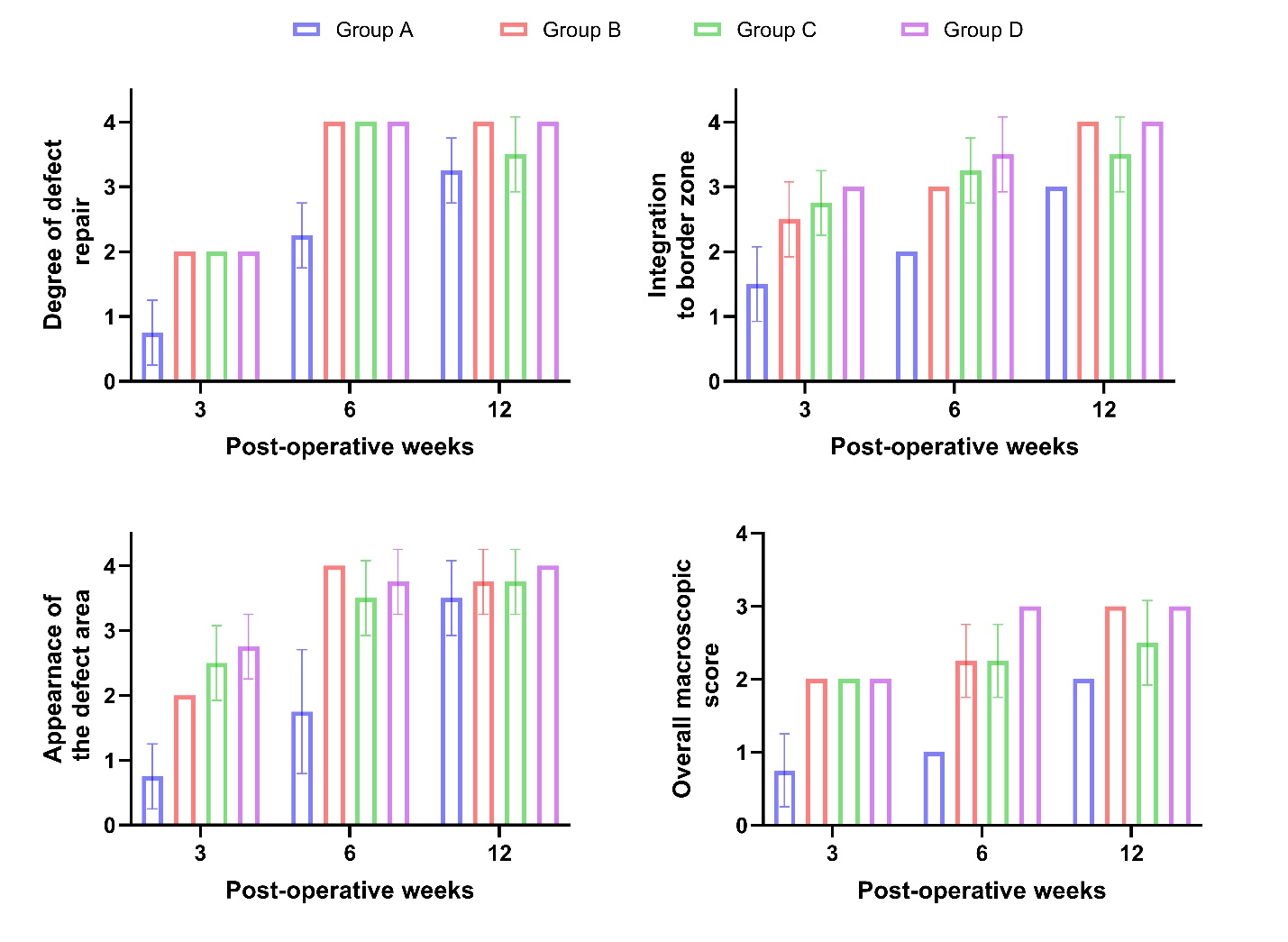


**Figure 2.** Charts of the ICRS gross evaluation score at 3, 6 and 12 weeks postoperative showed significant increase in the degree of defect repair, integration to border zone, appearance of the defect area and overall gross observation score in group D compared with group A B and C. Group A: Control Group, Group B: PRF, Group C: MNCs, Group D: PRF and MNCs.


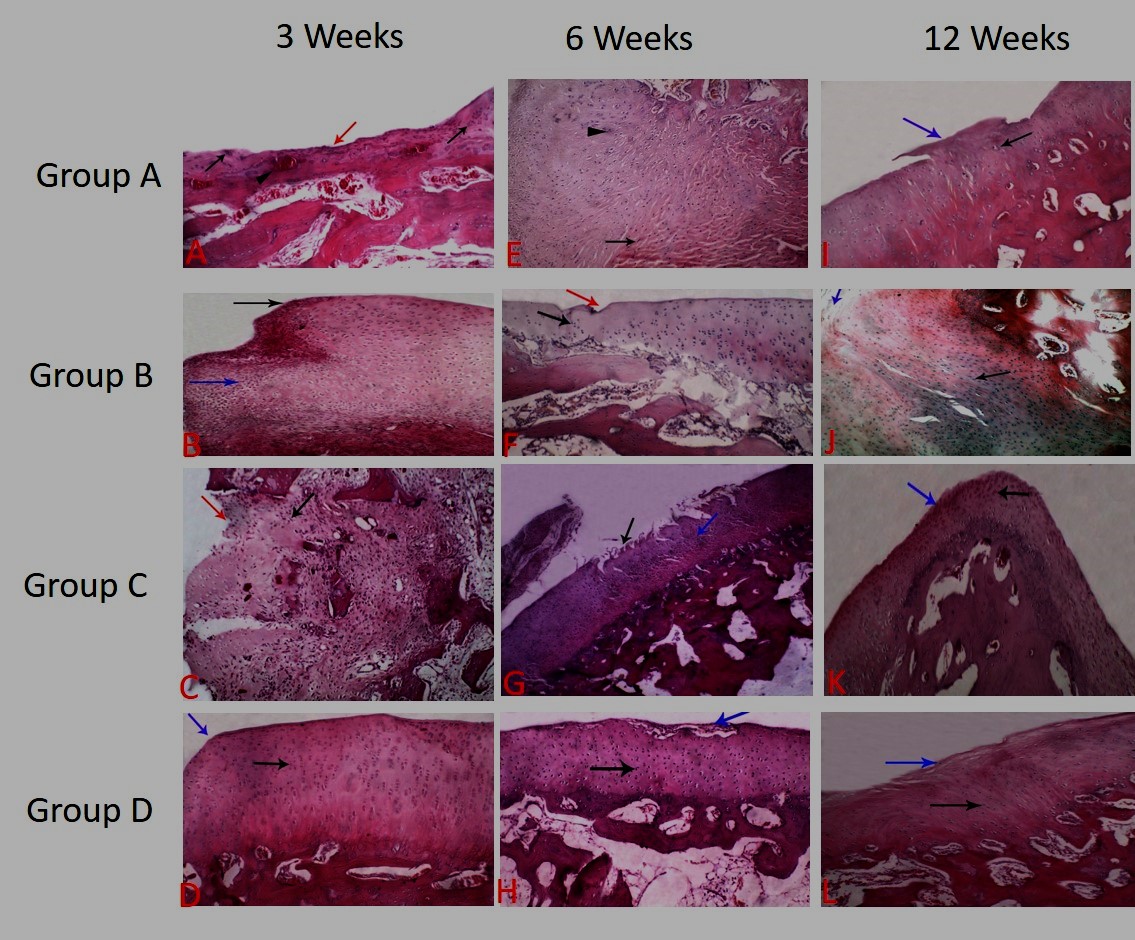


Figure 3: Histopathological view of osteochondral defect at trochlear groove of left stifle joint of white New Zealand rabbit at 3, 6 and 12 weeks postoperative A, Erosion of articular surface with exposing subchondral bone (red arrow) with aggregation of chondrocytes in the form of clusters at the edges of osteochondral defect (black arrows) and congestion of subchondral capillaries (arrow head) in group A at 3 week. B, Proliferation of the fibrocartilagenous tissue filling the osteochondral defect (black arrow) characterized disorganized chondrocytes in the form of clusters and columnar, and spindle shaped fibroblasts (blue arrow) in group B at 3 week. C, Fibrocollagenous tissue (red arrow) filling the osteochondral defect characterized by aggregated chondrocytes and spindle shaped fibroblasts in group C (black arrow) at 3 weeks. D, Smooth incontinuous surface (blue arrow)with fibrocartilage filling the osteochondral defect characterized by mixed columnar and clustered shaped chondrocytes ( black arrow) in group D at 3 week.E, Exuberant proliferation of fibrocartilagenous tissue filling the osteochondral defect (arrow) and aggregation of chondrocytes in the form of clusters at the edges (arrow head) in group A at 6 weeks. F, Displays smooth incontinuous surface (red arrow) with hyaline cartilage filling the osteochondral defect characterized by columnar shaped chondrocytes (black arrow) in group B at 6 week. G showed Cartilage fibrillation with irregular surface (black arrow) with fibrocartilage filling the osteochondral defect characterized by mixed columnar and clustered shaped chondrocytes ( blue arrow) in group C at 6 week. Figure H showed Smooth incontinuous surface (blue arrow) with hyaline cartilage filling the osteochondral defect characterized by columnar shaped chondrocytes ( black arrow) in group D at 6 week. Figure I showed : Fibrocartilagenous tissue filling the osteochondral defect (blue arrow) characterized by hypocellualrity and aggregation of chondrocytes in the form of clusters (black arrow) in group A at 12 week. Figure J showed Smooth continuous surface (blue arrow) with hyaline cartilage filling the osteochondral defect characterized by columnar shaped chondrocytes ( black arrow) in group B at 12 week. figure K showed Smooth continuous surface (blue arrow)with mixed hyaline and fibrocartilage cartilage filling the osteochondral defect characterized by mixed columnar and clustered shaped cells (black arrow) in group C at 12 week . Figure L showed Smooth continuous surface (blue arrow)with hyaline cartilage filling the osteochondral defect characterized by columnar shaped chondrocytes ( black arrow) in group D at 12 week. Group A: Control Group, Group B: PRF, Group C: MNCs, Group D: PRF and MNCs


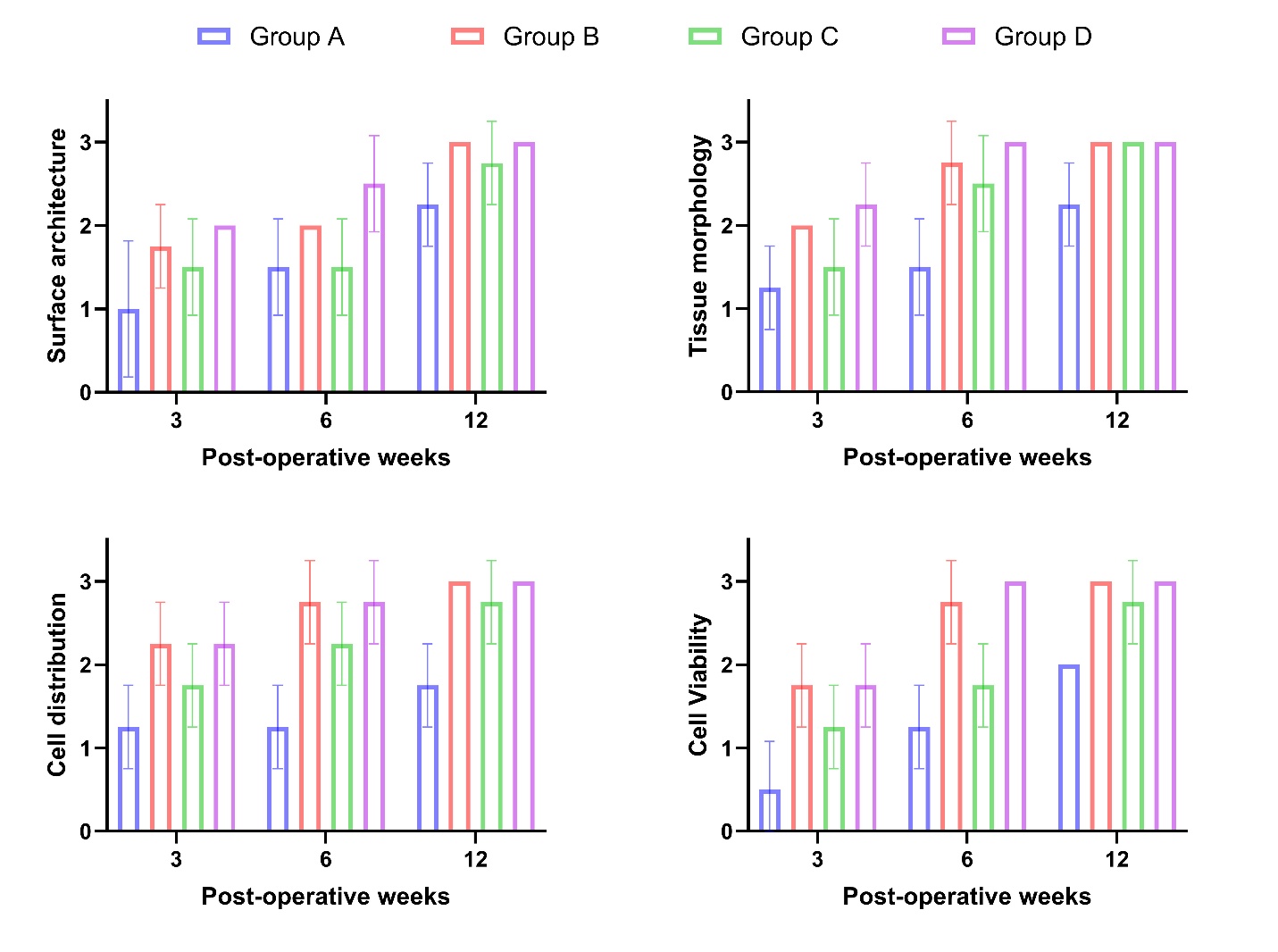


**Figure 4.** Chart of the ICRS histological score at 3, 6 and 12 weeks postoperative showing significant increase in tissue morphology, tissue architecture, cell distribution and cell viability in group D compared with other groups. Group A: Control Group, Group B: PRF, Group C: MNCs, Group D: PRF and MNCs


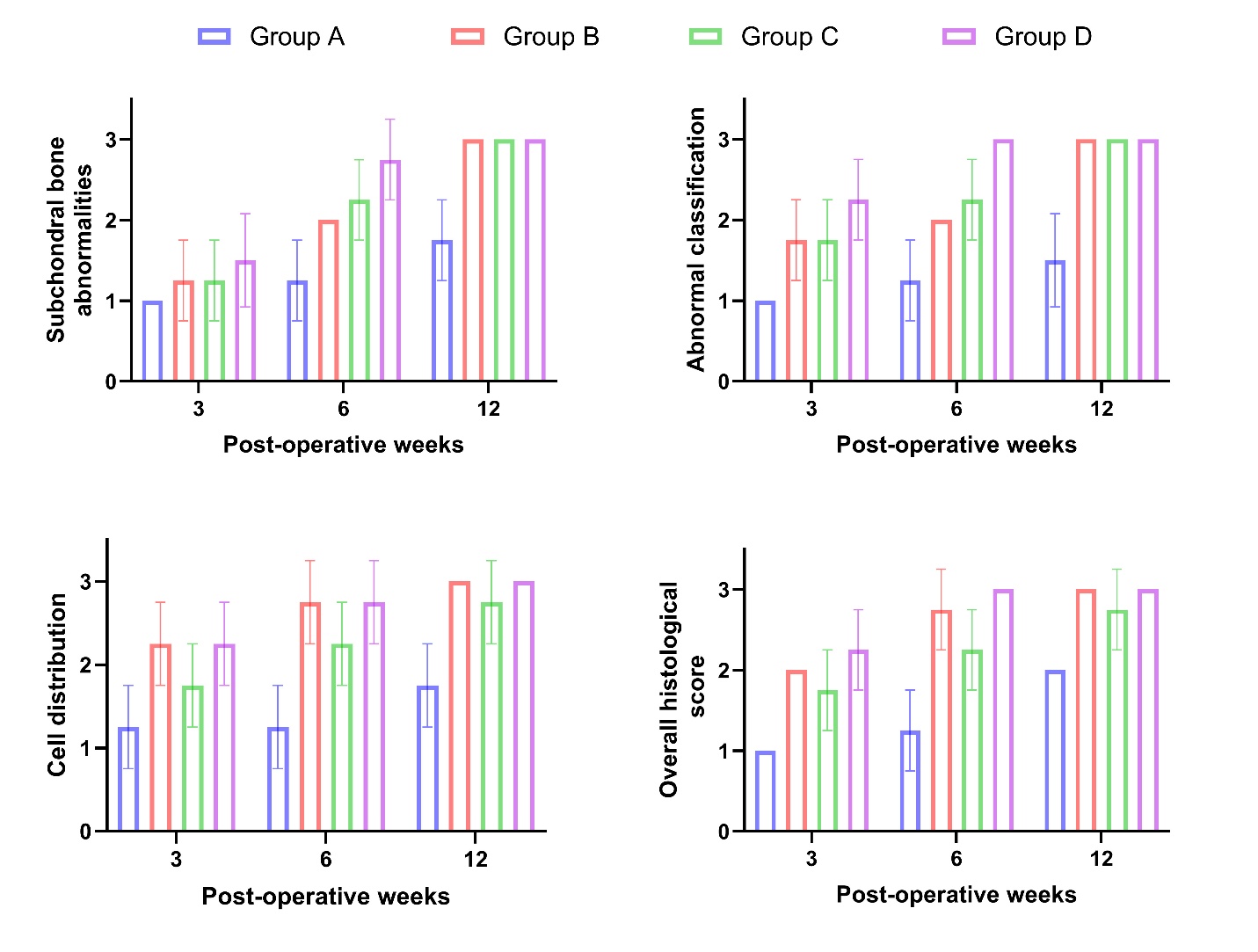


**Figure 5**. Chart of the ICRS histological score at 3, 6 and 12 weeks postoperative showing significant increase in the score of subchondral bone abnormalities, abnormal calcification, safranin O staining and overall histological evaluation in group D compared with other groups. Group A: Control Group, Group B: PRF, Group C: BMNCs, Group D: PRF and BMNCs.


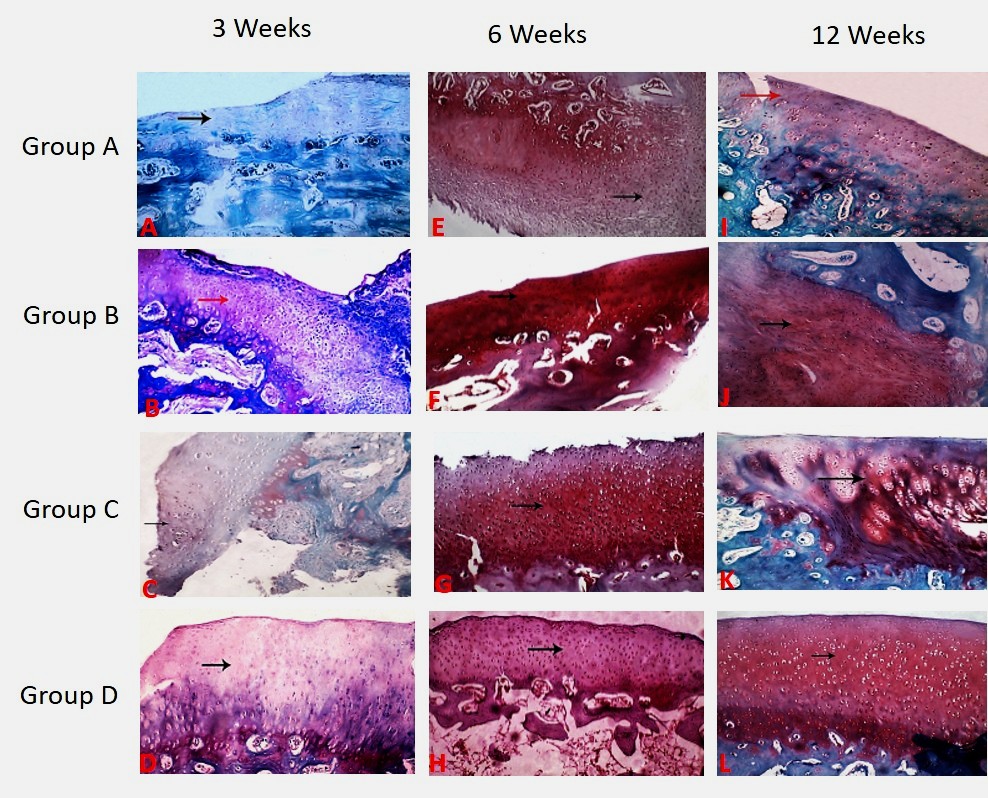


Figure 6: Histopathological view of osteochondral defect at trochlear groove of left stifle joint of white New Zealand rabbits at 3, 6 and 12 weeks postoperative showed normal matrix staining by safranin O (red color) and chondrocytes arrangement in group D followed by groups B, C, and A respectively (safranin O stain, 100x).Group A: Control Group, Group B: PRF, Group C: BMNCs, Group D: PRF and BMNCs


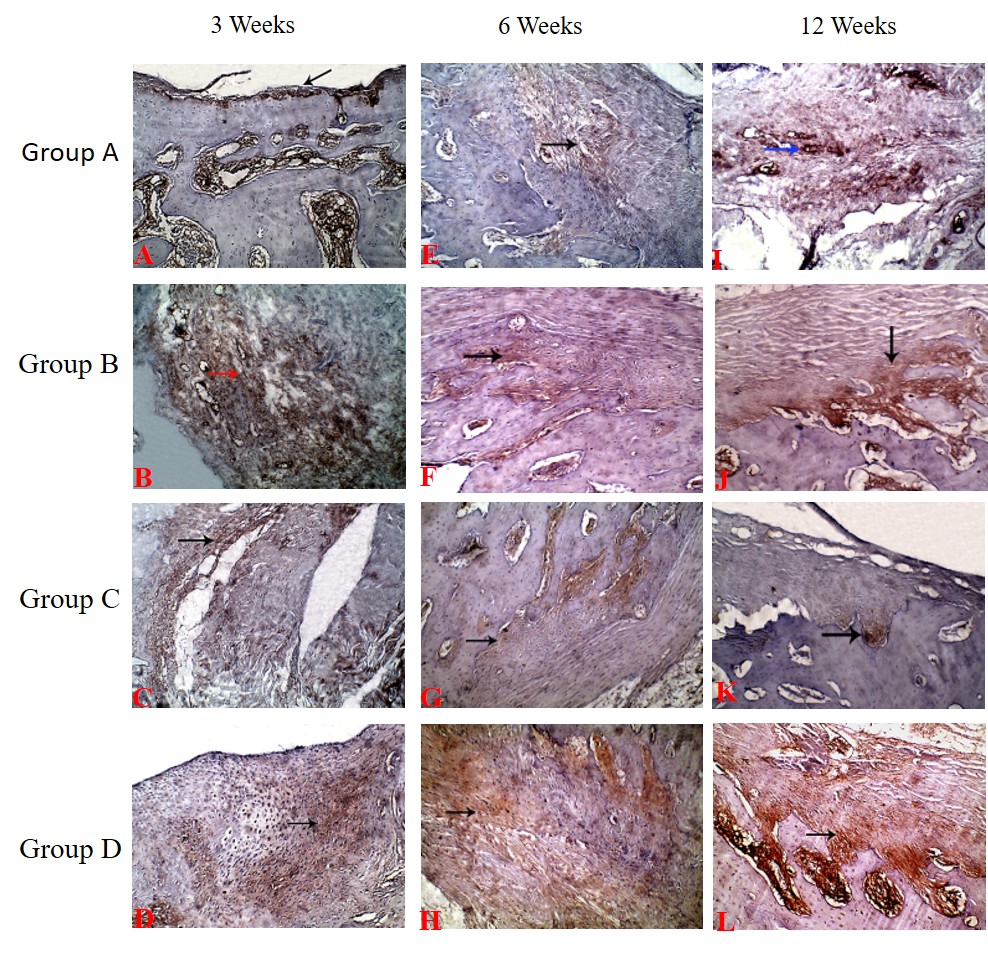


**Figure 7:** Immunohistochemical view of the defects at 3, 6 and 12 weeks postoperative (IHC, 100x). Group A: Control Group, Group B: PRF, Group C: BMNCs, Group D: PRF and BMNCs.


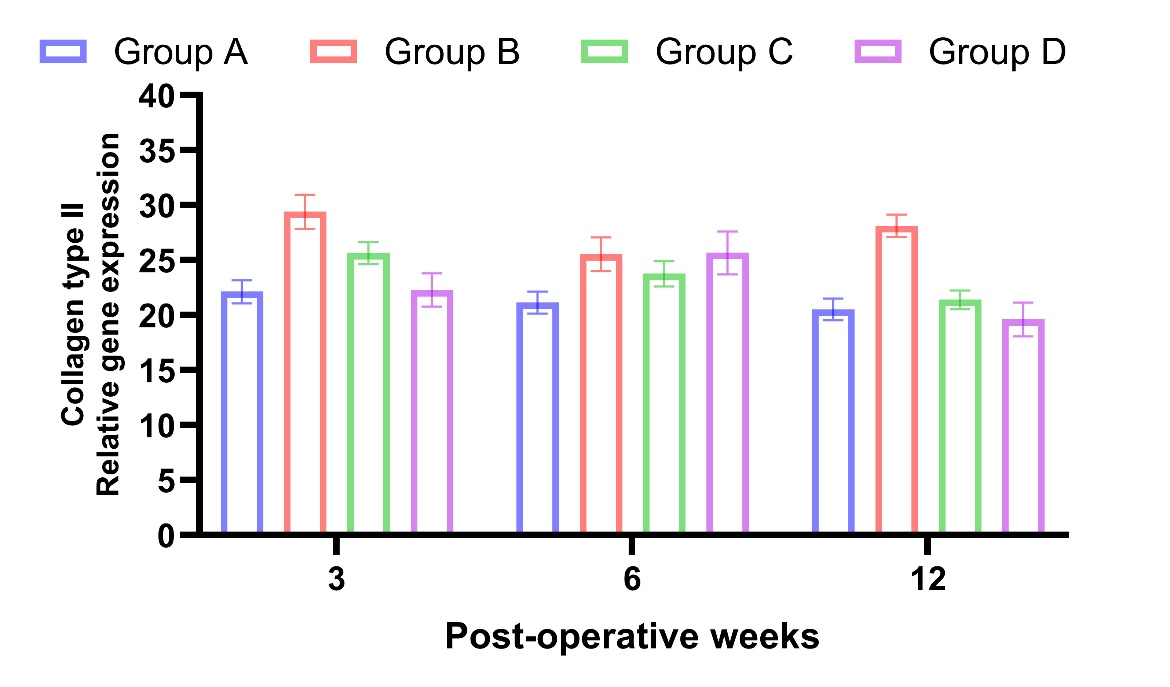


**Figure 8:** Chart illustrates the relative gene expression of Collagen type II at 3, 6 and 12 weeks postoperative in regenerated tissue following repair of osteochondoral defect in stifle joint of rabbits. Group A: Control Group, Group B: PRF, Group C: BMNCs, Group D: PRF and BMNCs.


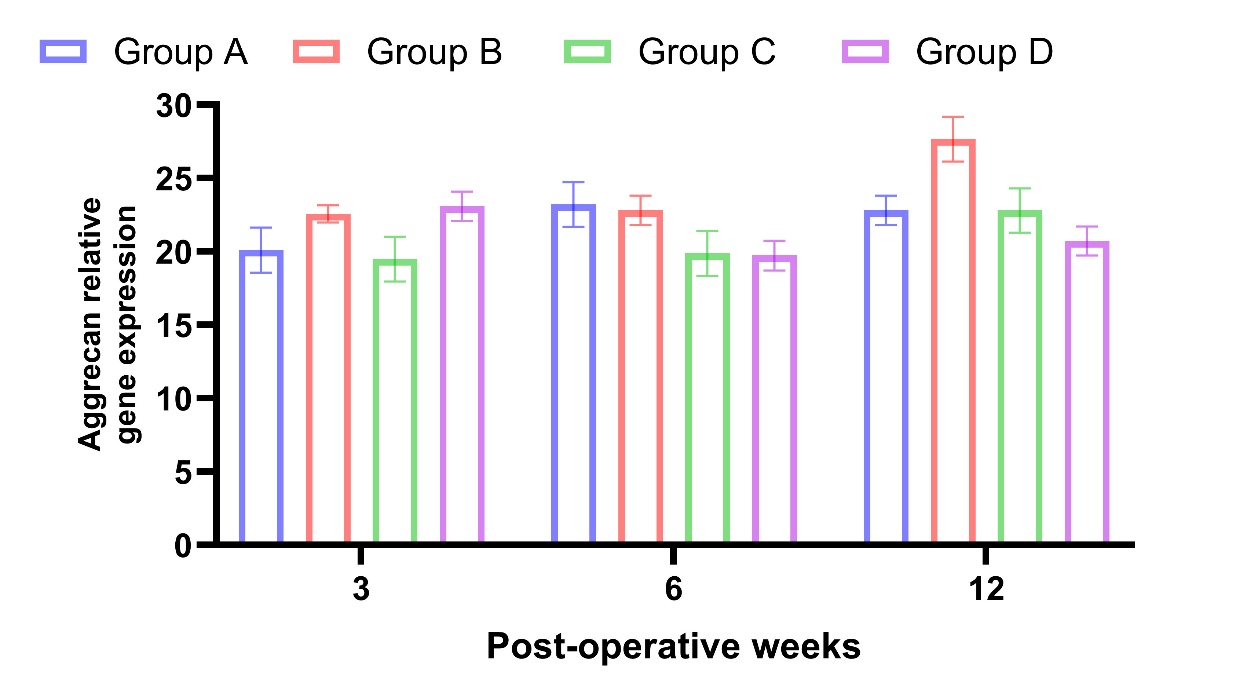


**Figure 9:** Chart illustrates the relative gene expression of aggrecan at 3, 6 and 12 weeks postoperative in regenerated tissue following repair of osteochondoral defect in stifle joint of rabbits. Group A: Control Group, Group B: PRF, Group C: BMNCs, Group D: PRF and BMNCs
